# A neurochemical map of the developing amphioxus nervous system

**DOI:** 10.1186/1471-2202-13-59

**Published:** 2012-06-07

**Authors:** Simona Candiani, Luca Moronti, Paola Ramoino, Michael Schubert, Mario Pestarino

**Affiliations:** 1 Dipartimento per lo Studio del Territorio e delle sue Risorse, Università di Genova, Viale Benedetto XV, 5, 16132 Genoa, Italy; 2 Institut de Génomique Fonctionnelle de Lyon (UCBL, CNRS UMR5242, ENSL, INRA 1288), Ecole Normale Supérieure de Lyon, 46 allée d'Italie, 69364, Lyon Cedex 07, de Lyon, France

**Keywords:** *Branchiostoma*, Cephalochordate, Chordate evolution, Lancelet, Neural patterning, Neurotransmitter

## Abstract

**Background:**

Amphioxus, representing the most basal group of living chordates, is the best available proxy for the last invertebrate ancestor of the chordates. Although the central nervous system (CNS) of amphioxus comprises only about 20,000 neurons (as compared to billions in vertebrates), the developmental genetics and neuroanatomy of amphioxus are strikingly vertebrate-like. In the present study, we mapped the distribution of amphioxus CNS cells producing distinctive neurochemicals. To this end, we cloned genes encoding biosynthetic enzymes and/or transporters of the most common neurotransmitters and assayed their developmental expression in the embryo and early larva.

**Results:**

By single and double in situ hybridization experiments, we identified glutamatergic, GABAergic/glycinergic, serotonergic and cholinergic neurons in developing amphioxus. In addition to characterizing the distribution of excitatory and inhibitory neurons in the developing amphioxus CNS, we observed that cholinergic and GABAergic/glycinergic neurons are segmentally arranged in the hindbrain, whereas serotonergic, glutamatergic and dopaminergic neurons are restricted to specific regions of the cerebral vesicle and the hindbrain. We were further able to identify discrete groups of GABAergic and glutamatergic interneurons and cholinergic motoneurons at the level of the primary motor center (PMC), the major integrative center of sensory and motor stimuli of the amphioxus nerve cord.

**Conclusions:**

In this study, we assessed neuronal differentiation in the developing amphioxus nervous system and compiled the first neurochemical map of the amphioxus CNS. This map is a first step towards a full characterization of the neurotransmitter signature of previously described nerve cell types in the amphioxus CNS, such as motoneurons and interneurons.

## Background

Although the genetic and developmental mechanisms of nervous system organization in vertebrates have attracted considerable attention, relatively little is known about the evolutionary origins of the vertebrate central nervous system (CNS). In all chordates, which, in addition to vertebrates, comprise the tunicates and cephalochordates (amphioxus), the anterior end of the dorsal, hollow nerve cord is enlarged to form a (at least diencephalic) forebrain, a possible midbrain and a hindbrain [1-5]. However, a definite midbrain and a midbrain–hindbrain organizer may have been vertebrate innovations [2,6].

**Figure 1:**
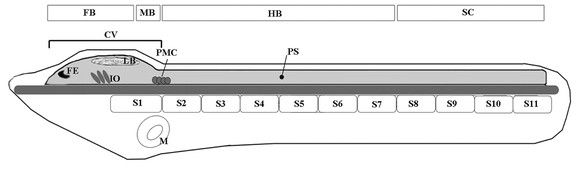
**Schematic representation of an amphioxus early larva. **The central nervous system (CNS) of an amphioxus early (36-hr) larva includes an anterior cerebral vesicle (CV), which, anteriorly, contains a frontal eye complex (FE) and, posteriorly, the infundibular organ (IO) as well as the lamellar body (LB). At the posterior end of the CV, there is a small midbrain-like region that includes the tectal zone extending from the posterior of the lamellar body to the anterior of the primary motor center (PMC). Posterior to the CV, the amphioxus CNS is divided into a hindbrain-like region, (extending from the posterior part of the PMC to the boundary of somites 7 and 8) and a territory corresponding to the vertebrate spinal cord. FB, forebrain; MB, midbrain; HB, hindbrain; SC, spinal cord; PS, first pigment spot; S, somite; N, notochord; M, mouth.

Amphioxus, with its vertebrate-like body plan and unduplicated genome [7,8], is a good model system to study CNS development and neuronal cell type diversity. The amphioxus CNS, which is composed of about 20,000 neurons, consists of a cerebral vesicle, a swelling at the rostral end of the nerve cord that corresponds to the vertebrate diencephalon plus a short midbrain, a hindbrain and a spinal cord [9-13] (Figure 1). The gene networks for patterning the anteroposterior and dorsoventral axes of the neural tube are likely conserved between amphioxus and vertebrates [14-17]. Moreover, molecular and anatomical studies suggest that the amphioxus and vertebrate brain are characterized by several homologous structures: the frontal eye complex (homolog of the vertebrate lateral eyes), the lamellar body (homolog of the vertebrate epiphysis), and the infundibulum [12] (Figure 1). Unlike vertebrates, the neural tube of amphioxus does not possess morphologically segmented rhombomeres. Collinear expression of Hox genes as well as the segmental expression patterns of genes, such as *islet* and *ERR*, nonetheless suggest that the amphioxus CNS contains a regionalized hindbrain [16,18-20] and some authors have proposed that the establishment of this hindbrain organization requires signaling from the segmented mesoderm [20]. Neurotransmitter chemistry has been relatively conserved through metazoan evolution, while brain size and cytoarchitecture have been altered considerably in different lineages, particularly in vertebrates. Therefore, amphioxus, with its simple vertebrate-like CNS, is a favorable model for studying the evolution of chordate and vertebrate neuroanatomy and neurophysiology. In a previous study, we have identified a gene encoding amphioxus tyrosine hydroxylase (*AmphiTH*), the rate-limiting enzyme for dopamine (DA) biosynthesis and studied the localization of dopaminergic neurons during amphioxus development [21]. Similarly, we have studied the appearance of cholinergic neurons by assessing the expression of choline acetyltransferase (*ChAT*) and vesicular acetylcholine transporter (*VAChT*) [22]. By double whole-mount in situ hybridization, we demonstrated that *VAChT* and *ChAT* are coexpressed in the same cells of the amphioxus neural tube. Moreover, both amphioxus *VAChT* and *ChAT* are alternative transcripts generated from a single gene locus (cholinergic gene locus or CGL) [22]. Moreover, the serotonergic system has been investigated by immunocytochemistry in amphioxus embryos and larvae [23,24]. 

Here, we use neurotransmitter markers in amphioxus to reconstruct a neurochemical map of the developing cephalochordate nervous system. Using vesicular transporters and key enzymes for neurotransmitter biosynthesis as markers, we assessed the neuronal differentiation in the developing amphioxus nervous system focussing on the time of appearance of glutamatergic, GABAergic, glycinergic, cholinergic and serotonergic neurons. Although we are aware that physiological and pharmacological assays are required to definitively demonstrate the utilization of a specific neurotransmitter by a given amphioxus neuron, our work nonetheless revealed the distribution of neuronal cell bodies engaged in synthesizing particular neurochemicals in the developing amphioxus CNS and allowed us to construct the first neurochemical map of the amphioxus embryo.

## Results

In order to classify the neurons of the developing amphioxus CNS on the basis of their neurotransmitter properties, we cloned genes encoding vesicular transporters and/or key enzymes for transmitter synthetic pathways and studied their expression by whole mount in situ hybridization (Table 1). Glutamatergic neurons were identified by using as marker the vesicular glutamate transporter (VGLUT), which in vertebrates is required for glutamate accumulation and transmission. In the genome of the Florida amphioxus (*Branchiostoma floridae*), we found four potential VGLUT-related sequences (Additional file 1). Phylogenetic analyses established that only one of the four sequences is a true ortholog of the three vertebrate VGLUT sequences (Additional file 1 and Additional file 2). This amphioxus *VGLUT* gene was subsequently isolated by PCR and used to synthetize the riboprobe for in situ hybridization.

**Table 1:** List of the genes, encoding vesicular transporters and/or key enzymes for neutransmitter biosynthesis, studied in the present work

**Neurotransmitter**	**Transporter**	**Biosynthetic enzyme**
Glutamate	Vesicular Glutamate Transporter (VGLUT)	
Serotonin (5-HT)	Serotonin Transporter (SERT)	Tryptophan Hydroxylase (TpH)
GABA	Vesicular GABA/Glycine Transporter (VGAT)	Glutamic Acid Decarboxylase (GAD*)*
Glycine	Vesicular GABA/Glycine Transporter (VGAT)	
Acetylcholine (ACh)	Vesicular Acetylcholine Transporter (VAChT)	

Amphioxus possesses at least one ortholog of the mammalian genes encoding tryptophan hydroxylase (TpH), the rate-limiting enzyme in serotonin synthesis and a serotonin transporter (SERT) acting as both symporter and antiporter in the presynaptic membrane (Additional file 1 and Additional file 2). Amphioxus *TpH* and *SERT* served as molecular markers for locating the serotonin-producing neurons in the developing amphioxus nervous system. Both markers show the same expression pattern and are likely expressed at very low levels, because, compared to the other neurotransmitter markers used in the present work, both required an extended development time with the alkaline phosphatase-mediated detection system. We were thus unable to obtain very strong signals in our double in situ hybridization experiments with these two markers.

To follow the differentiation of GABAergic neurons in amphioxus, we studied the expression of glutamic acid decarboxylase (GAD*),* the rate-limiting enzyme in GABA biosynthesis. Moreover, we simultaneously assessed the developmental expression of the vesicular GABA/glycine transporter (VGAT) that is responsible for the uptake into the synaptic vesicle of both GABA and glycine [25]. We found that amphioxus possesses single genes encoding *GAD* and *VGAT* (Additional file 1 and Additional file 2), both of which were cloned by PCR. However, since *VGAT* is expressed in both GABAergic and glycinergic neurons [25], in order to identify the glycinergic neurons in the amphioxus CNS, we needed to perform double in situ hybridization experiments with both *GAD* and *VGAT*. Thus, GABAergic neurons are *VGAT*-positive and *GAD*-positive, while glycinergic neurons are *VGAT*-positive and *GAD*-negative.

**Figure 2:**
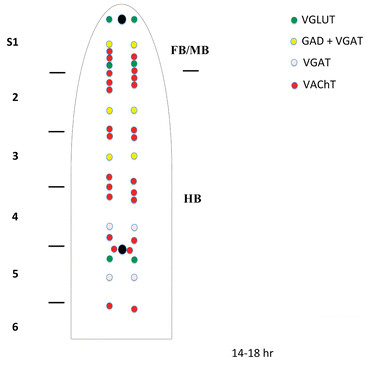
**Schematic representation of the neural tube of amphioxus 14–18 hr neurulae showing expression of genes involved in the synthesis and/or transport of neurotrasmitters. **The neural tube (anterior on top) has been opened such that cells located dorsally in the neural tube are lateral in the diagram. Black circles = location of the frontal eye (anterior) and of the first pigment spot (poterior). S1-6 = position of somites 1–6. FB/MB = forebrain and midbrain; HB = hindbrain. The spinal cord starting at the level of somite 7 is not shown due to the lack of labelled neurons at these stages.

**Figure 3:**
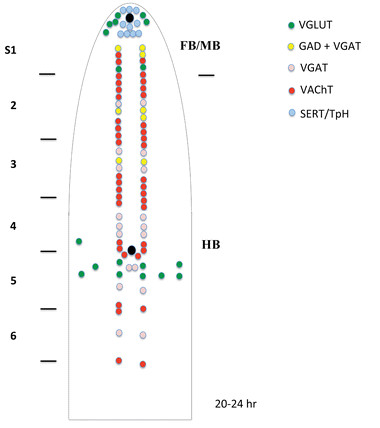
**Schematic representation of the neural tube of amphioxus 20–24 hr neurulae of showing expression of genes involved in the synthesis and/or transport of neurotrasmitters. **The neural tube (anterior on top) has been opened such that cells located dorsally in the neural tube are lateral in the diagram. Black circles = location of the frontal eye (anterior) and of the first pigment spot (poterior). S1-6 = position of somites 1–6. FB/MB = forebrain and midbrain, HB = hindbrain. The spinal cord starting at the level of somite 7 is not shown due to the lack of labelled neurons at these stages.

Finally, we used double in situ hybridization to obtain more synoptic maps of neurochemically-marked neurons (Figures 2 and 3). In addition to *VGAT* and *VGLUT*, we also included the gene encoding the amphioxus vesicular acetylcholine transporter (*VAChT*) as marker for cholinergic neurons. We hence analyzed embryos double-stained for *VGAT* and *VGLUT*, *VGLUT* and *VAChT* as well as *VGAT* and *VAChT*.

### Glutamatergic neurons

**Figure 4:**
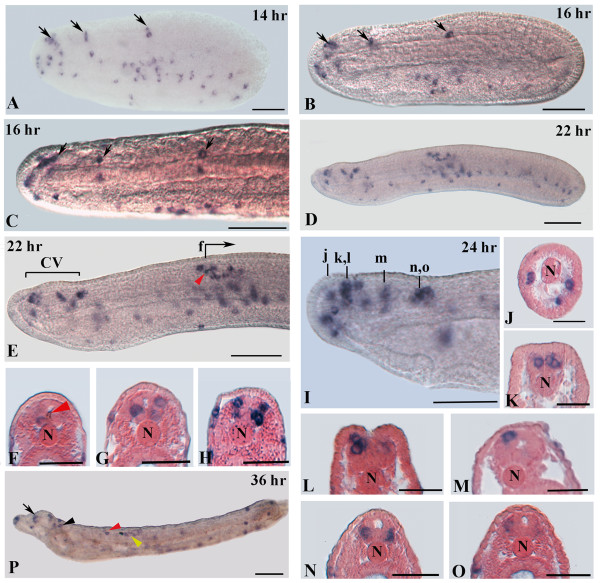
**VGLUT expression in developing amphioxus embryos. **Whole mounts with anterior to the left (scale bars = 50 μm) and cross-sections (scale bars = 25 μm) viewed from the caudal end of the animal. **A:** 14-hr neurula showing expression in single ectodermal cells in the ventrolateral region and in three spots of the neural tube (arrows). **B:** 16-hr neurula with expression in three spots of the neural tube (arrows). **C:** Dorsal view of the embryo in B showing three pairs of VGLUT positive neurons in the neural tube (arrows). **D:** Side view of a 22-hr neurula. **E:** Magnification of the anterior part of the embryo in D, showing expression in three cell clusters of the cerebral vesicle (CV) and in several neurons located in close proximity to the first pigment spot. Labeled ectodermal sensory cells are conspicuous near the first pigment spot. **F-H:** Consecutive transverse sections starting at the level of f in E. **I:** Anterior end of a 24-hr larva. **J-O:** Transverse sections at levels indicated in I. **P:** 36-hour larva. Several labeled sensory cells are visible along the body of the larva and at the tip of the rostrum. VGLUT transcripts are also detectable in cells of the frontal eye complex (arrow), in neurons of the posterior cerebral vesicle (black arrowhead) and in cells of the neural tube anterior (red arrowhead) and posterior (yellow arrowhead) to the first pigment spot. N, notochord. The first pigment spot in E, N and P is indicated by a red arrowhead.

Expression of amphioxus *VGLUT* is first detectable in the CNS of the 14-hr neurula in three pairs of cells located, respectively, in the anterior tip of the CNS, at posterior end of the cerebral vesicle and in close proximity of the first pigment spot (Figures 2 and 4A). At this stage, *VGLUT* is also expressed in ectodermal cells located at the anterior tip of the embryo and ventrolaterally on both sides of the embryo (Figure 4A). The latter ectodermal cells probably correspond to type 1 ectodermal sensory cells [26,27], which originate ventrally and migrate dorsally during the neurula stage. *VGLUT* expression remains essentially unchanged in the 16-hr neurula (Figures 2, 4B and C). At 22 hrs and 24 hrs, however, additional cells in the anterior CNS begin to express *VGLUT*, including cells located dorsolaterally in the anterior tip of the cerebral vesicle (Figures 3 and 4D-O). *VGLUT* expression is also found in dorsolateral and ventral cell bodies in proximity to the first pigment spot (Figures 3 and 4E-H). Moreover, scattered ectodermal cells along both flanks of the body are still labeled (Figure 4D-H). At 36 hrs, *VGLUT* transcription is initiated in the neural tube just anterior to the first pigment spot (Figure 4P). In 36-hr larvae, *VGLUT*-positive cells are also detectable in the anterior tip of the CNS at level of the frontal eye, in the posterior cerebral vesicle, in the neural tube anterior and posterior to the first pigment spot and in ectodermal sensory cells (Figure 4P). 

### Serotonergic neurons

**Figure 5:**
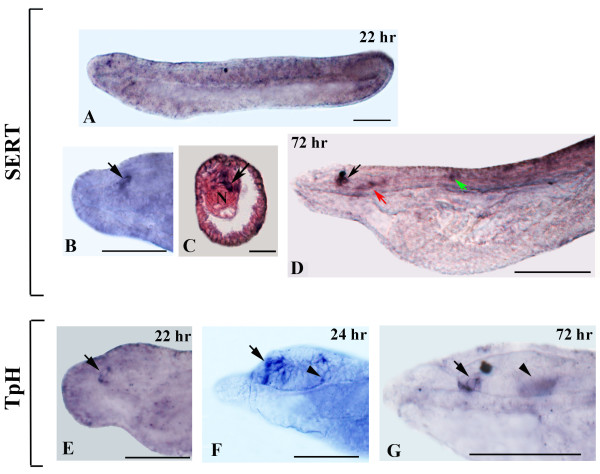
***TpH *and *SERT *expression during amphioxus development. **Whole mounts with anterior to the left. The cross-section in **C **is viewed from the caudal end of the animal. Whole mount and section scale bars are 50 μm and 25 μm, respectively. **A: **Side view of a 22-hour embryo. **B: **Magnification of the anterior end of the embryo in **A **showing *SERT*-positive cells in the most anterior tip of the neural tube (arrow). **C: **Cross-section through the most anterior labeled cells in **B **(arrow). **D: **Anterior portion of a 72-hour larva. Transcripts are detectable in cells of the frontal eye complex (black arrow), in the center and posterior end of the cerebral vesicle (red arrow) and in cells in the hindbrain region (green arrow). **E: **Anterior end of a 22-hr embryo showing *TpH *expression in the precursor region of the frontal eye complex (arrow). **F,G: ***TpH *expression in the anterior end of 24-hr and 72-hr larvae, respectively. *TpH *is expressed in cells of the frontal eye (arrow) and in ventrolateral cells of the cerebral vesicle (arrowhead). N, notochord.

*TpH*- and *SERT*-expressing neurons are located in 22-hr neurulae in the most anterior tip of the cerebral vesicle, where the frontal eye complex will develop (Figures 3, 5A-C and 5E). By 24-hr, *TpH*-positive neurons are also detectable in ventrolateral cells of the cerebral vesicle (Figures 3 and 5F). Expression is essentially unchanged in the 72-hr larva (Figures 5D and G), with the notable exception of a few *SERT*-positive neurons that become detectable in the hindbrain region (Figure 5D).

### GABAergic and glycinergic neurons

**Figure 6:**
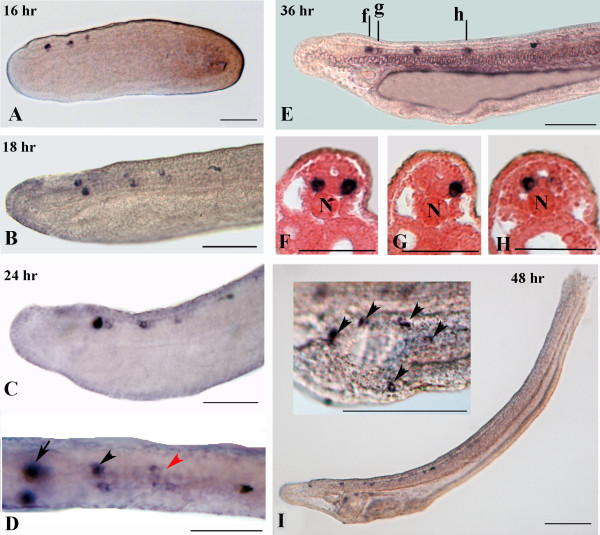
**Expression of *GAD *during amphioxus development. **Whole mounts with anterior to the left. The cross-sections in **F-H **are viewed from the caudal end of the animal. Whole mount and section scale bars are 50 μm and 25 μm, respectively. **A: **Side view of a 16-hr neurula showing three spots of expression in the neural tube. **B **Magnification of a 18-hr neurula showing three pairs of *GAD*-positive neurons. **C **Anterior region of a 24-hr larva. **D **Dorsal view of the embryo in **C **showing conspicuous expression in clustered neurons (arrow) at the posterior end of the cerebral vesicle. At more posterior levels of the neural tube, there are two more clusters of labeled neurons (black and red arrowheads). **E: **Lateral view of a 36-hour larva. **F-H: **Cross-sections of the larva shown in **E **through levels f, g and h, respectively. **I: **Side view of 48-hour larva. The inset shows *GAD*-positive sensory cells around the oral cavity (arrowheads). N, notochord.

*GAD* expression is first detectable in the 16-hr neurula in three pairs of cells in the neural tube: one pair in the cerebral vesicle and two pairs in the hindbrain region (Figures 2 and 6A). Expression is unchanged in the 18-hr neurula (Figure 6B). By 24-hr and 36-hr, the first two cell pairs have been replaced by two clusters of three cells each (Figures 3 and 6C-E). *GAD*-expressing cells are arranged ventrolaterally in the neural tube (Figure 6F-H). By 48-hr, excepting the appearance of *GAD*-positive neurons anterior and posterior to the first pigment spot, the expression of *GAD* in the neural tube remains unchanged. At this stage, ectodermal sensory neurons around the oral cavity also express *GAD* (Figure 6I).

**Figure 7:**
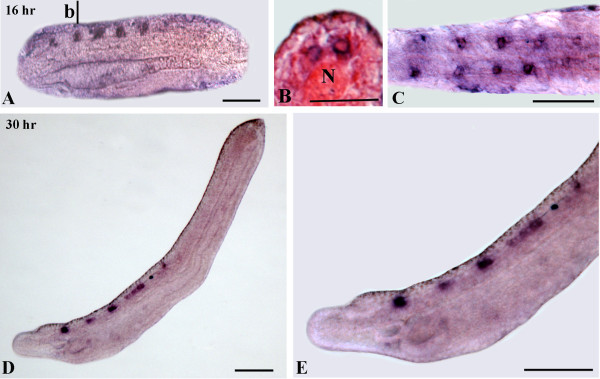
**Expression of *VGAT *during amphioxus development. **Whole mounts with anterior to the left. The cross-section in **C **is viewed from the caudal end of the animal. Whole mount and section scale bars are 50 μm and 25 μm, respectively. **A: **Side view of a 16-hour neurula showing five spots of *VGAT *expression in the neural tube. **B: **Cross-section of the embryo in **A **through level b. Two ventrolateral neurons are expressing *VGAT*. **C: **Enlarged dorsal view of the embryo in **A **showing five pairs of *VGAT*-positive neurons. **D: ** Side view of a 30-hour larva. **E: **Magnification of the larva in **D**. N, notochord.

**Figure 8:**
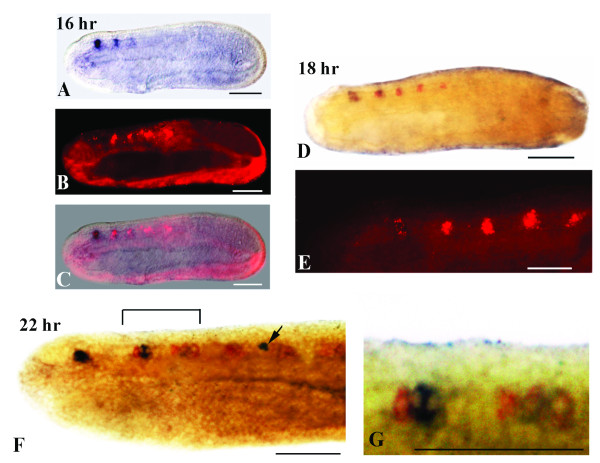
**Overlapping expression of *GAD *and *VGAT *revealed by double in situ hybridization. **Anterior is to the left and whole mount scale bars are 50 μm. **A-C: **Lateral views of a 16-hr neurula. **A: ***GAD *expression is detectable in three spots of the neural tube. **B: **Double-labeled embryo viewed by epifluorescence showing five pairs of *VGAT*-positive neurons. **C: ** Merger of **A **and **B **showing the colocalization of *GAD *and *VGAT *in three anterior pairs of neurons. **D,E: **Lateral views of a 18-hr neurula. **D: **Double-labeled embryo. The dark brown staining shows co-expression of *GAD *and *VGAT, *while the red staining shows only *VGAT *expression. **E: **Magnification of the double-labeled embryo in **F **showing *VGAT*-positive neurons in epifluorescence. **F,H: **Double-labeled 22-hr neurula. **F: **Magnification showing *GAD *and *VGAT *co-expression (dark brown) and *VGAT *expression (red). The arrow indicates the first pigment spot. **G: ** Magnification of the bracketed region in F.

Amphioxus *VGAT* expression is first observed in the 16-hr neurula in five pairs of cells (Figures 2 and 7A,C): one pair in the cerebral vesicle and four pairs, regularly spaced, in the hindbrain region. A cross-section shows expression of *VGAT* in the ventral neural tube, which is identical to the localization of *GAD*-positive neurons (Figure 7B). The overall expression pattern of *VGAT* is maintained at later developmental stages, but the total number of *VGAT*-positive cells in each of the five cluster pairs increases with time (Figures 7D and E). At the mid-neurula stage (16–18 hr), glycinergic neurons correspond to the two posterior pairs of *VGAT*-positive/*GAD*-negative cells (Figures 2 and 8A-E). By the late neurula stage (22-hr), moving from anterior to posterior, the second cluster of labeled cells (at the level of somite 2) contains a mixture of glycinergic and GABAergic cells, while the third cluster (at the center of the somite 3) is characterized by at least one pair of GABAergic and two pairs of glycinergic neurons (Figures 3 and 8F,G). Both the fourth cluster and the labeled cells posterior to the first pigment spot maintain only a glycinergic phenotype (Figure 3). The *VGAT* and *GAD* expression patterns observed in late neurulae are still present at larval stages (data not shown).

### Construction of a neurochemical map in amphioxus by double in situ hybridization

**Figure 9:**
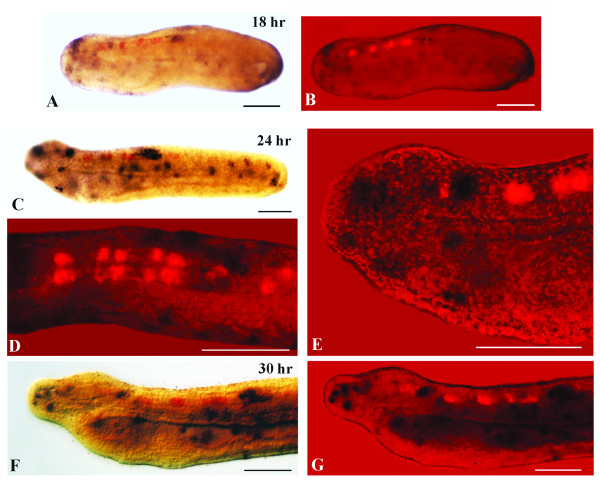
**Double in situ hybridization of *VGAT *and *VGLUT. ***Whole mounts with anterior to the left. Whole mount scale bars are 50 μm. In **A **, **C** and **F **the dark purple staining shows *VGLUT *expression, while the red staining shows *VGAT *expression. **B**, **D**, **E **and **G **show a superimposition of brightfield and epifluorescence with *VGLUT*- and *VGAT*-positive neurons. **A,B: **Side view of a 18-hr neurula. **C: **Side view of a 24-hr larva. **D: ** Magnified dorsal view of the larva shown in **C**. **E: **Magnified anterior part of the larva in **C**. **F,G: **Side view of the anterior third of a 30-hr larva.

**Figure 10:**
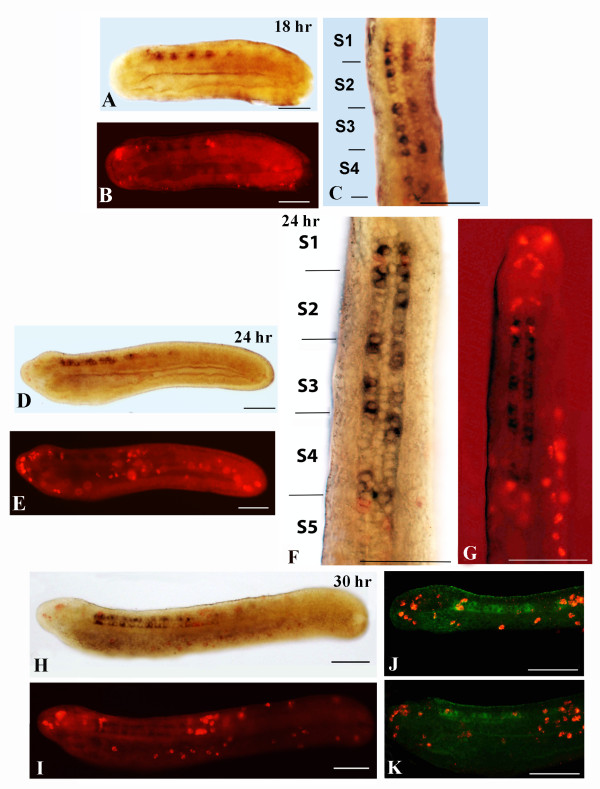
**Double in situ hybridization of *VAChT *and *VGLUT. ***Whole mounts with anterior to the left, except for **C**, **F **and **G**, where anterior is up. Whole mount scale bars are 50 μm. In **A**, **C**, **D, F **and **H**, the dark purple and red staining, respectively, show *VAChT *and *VGLUT * expression. In **B**, **E**, **G **and **I**, the superimposition of brightfield and epifluorescence shows *VAChT*- and *VGLUT*-positive neurons. **A,B: **Lateral views of a 18-hr neurula. **C: **Dorsal view of the embryo in **A **and **B**. **D,E: ** Lateral views of a 24-hr neurula. **F,G: **Dorsal views of the embryo in **D **and **E**. **H,I: **Lateral views of a 30-hr larva. **J,K: **Serial confocal fluorescence images of the embryo in **H **and **I**. S, somite.

**Figure 11:**
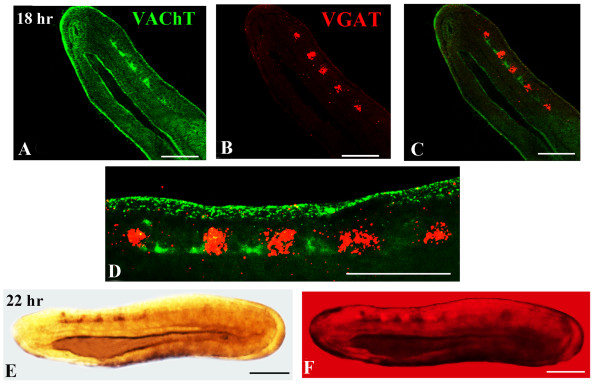
**Double in situ hybridization of *VAChT *and *VGAT. ***Whole mounts with anterior to the left and whole mount scale bars are 50 μm. **A-D: ** Serial confocal fluorescence images of a 18-hr neurula. **A: ***VAChT*-positive neurons (green). **B: ***VGAT*-positive neurons (red). **C: **Merger of **A **and **B**. **D: ** Magnification of the anterior neural tube shown in **C**. **E,F: **Lateral views of a 22-hr neurula. In **E**, the dark purple and red staining respectively show *VAChT *and *VGAT *expression. In **F**, the superimposition of brightfield and epifluorescence shows both *VAChT*-positive and *VGAT*-positive neurons.

We found that, at the neurula stage (14 hrs to 18 hrs), the amphioxus CNS contains different types of neurons. In the cerebral vesicle (the equivalent of the vertebrate forebrain/midbrain), we found two pairs of *VGLUT*-positive cells, one located anteriorly and one located posteriorly (Figures 2 and 9A,B), and one pair of *GAD/VGAT*-positive cells located in the center of the cerebral vesicle (Figures 2 and 8A,D). Moreover, in the 14–18 hr neurula, there is also one pair of *VAChT*-positive cells detectable in the cerebral vesicle (Figures 2 and 10A-C). The more posterior regions of the neural tube, starting at the level of somite 2 and extending to the boundary of somites 4 and 5, is characterized by a mixture of *VAChT*-labeled neurons and *GAD*/*VGAT*-positive (at the level of somites 2 and 3) or *VGAT*-positive/*GAD*-negative (at the level of somite 4) neurons (Figures 2, 8A-E and 11A-D). Importantly, the *VAChT*-positive and *GAD*-/*VGAT*-positive or *VGAT*-positive/*GAD*-negative neurons are organized into alternating clusters of cells (Figures 2, 8A-E and 11A-D). At the junction between somites 4 and 5, which corresponds to the region of the neural tube where the first pigment spot is forming, *VAChT*-, *VGLUT*- and *VGAT*-expressing neurons are organized into an anteroposterior array of cells (Figures 2, 9A and B, 10A and C, 11A-D).

These expression patterns are maintained in later neurula stages (20 hrs to 24 hrs) (Figures 3, 9C-E, 10D-G and 11E,F), with the notable exception of the appearance of two groups of *VGLUT*-positive neurons: one located dorsally in the anterior cerebral vesicle (Figures 3, 9C and E, 10D,E,G) and one located posterior to the first pigment spot (Figures 3 and 10E-G). Moreover, we observed that the total number of neurons constituting the *VGAT* and *VAChT* clusters increased at these later stages (Figures 3, 9C-E, 10D-G). At larval stages, there is no substantial difference in the basic organization of neuronal cell types in the amphioxus CNS except for an additional increase in the total number of neurons constituting the *VGAT* and *VAChT* clusters (Figures 9F and G, 10H-K).

Taken together, our in situ hybridization experiments yielded a neurochemical map of the developing amphioxus CNS. Surprisingly, this map suggests that, at least in the developmental stages examined, neurotransmitters do not colocalize in the amphioxus CNS. This finding contrasts with some examples of neurochemical colocalization observed in mollusks and vertebrates [28-30].

## Discussion

**Figure 12:**
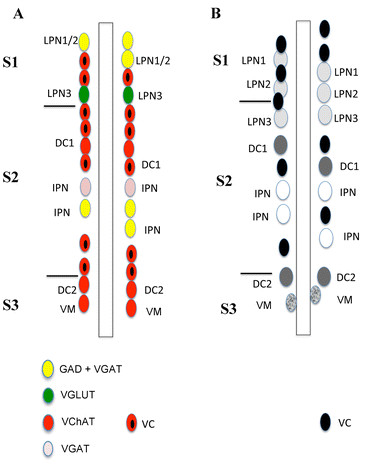
**Comparison of neurotransmitter cell types in amphioxus embryos with the distribution of ventrolateral neurons in amphioxus larvae. A: **Neurochemical map of amphioxus 20–24 hr embryos. **B: **Distribution of ventrolateral neurons in amphioxus 8–12.5 day larvae. Anterior is up and neuronal distribution is indicated only until the level of somite 3. Boundaries between somites (S) are used to indicate the relative position of different cell types in the neural tube. Motoneuron populations include dorsal compartment motoneurons (DC), ventral compartment motoneurons (VC) and visceral motoneurons (VM). Interneuron populations include the large paired neurons (LPN) and the ipsilateral projection neurons (IPN).

**Figure 13:**
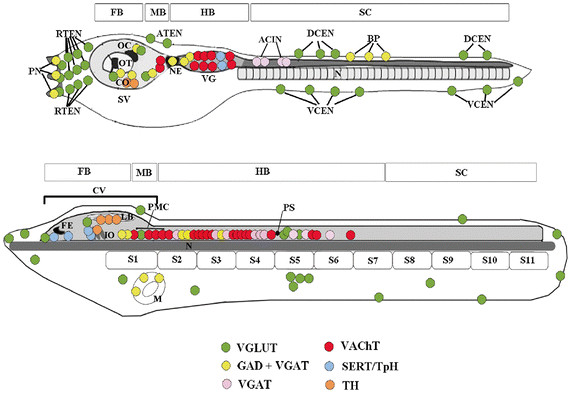
**Schematic representation of VGLUT, GAD + VGAT, VGAT, VAChT, SERT/TpH and TH expression in the central and peripheral nervous systems of the ascidian tunicate tadpole and the amphioxus larva. ** For both the ascidian tunicate tadpole and the amphioxus larva the regions of the central nervous system homologous to the vertebrate forebrain (FB), midbrain (MB), hindbrain (HB) and spinal cord (SC) are indicated. The central nervous system of the ascidian tunicate tadpole larva is divided into a sensory vesicle (SV), a visceral ganglion (VG), and a caudal nerve cord [36]. A slender neck (NE) region is present between the SV and VG. The anterior part of SV contains two sensory organs: the otolith (OT), used for the perception of gravity, and the ocellus (OC), for light reception. The left side of SV contains other sensory cells named coronet cells (CO). The VG consists of at least 8 motoneurons and some interneurons, while the anterior nerve cord contains the anterior caudal inhibitory neurons (ACIN). The ascidian tunicate tadpole peripheral nervous system consists of an adhesive organ with papillar neurons (PN), ectodermal neurons in the head (RTEN, rostral trunk epidermal neurons), trunk (ATEN, apical trunk epidermal neurons) and tail (DCEN and VCEN, dorsocaudal and ventrocaudal epidermal neurons, respectively), and bipolar interneurons (BP). The CNS of an amphioxus larva includes an anterior cerebral vesicle (CV), which, anteriorly, contains a frontal eye complex (FE) and, posteriorly, the infundibular organ (IO) as well as the lamellar body (LB). The primary motor center (PMC) is located at the posterior end of the CV and thus significantly anterior to the first pigment spot (PS). S, somite; N, notochord; M, mouth.

In the present work, we have studied the development of specific neuronal groups defined by their neurotransmitter phenotype in the amphioxus *B. floridae* and have compiled a synoptic neurochemical map of the developing amphioxus nervous system (Figures 12 and 13). This is an advance over previous studies of amphioxus neurons, which were limited to elucidating only one neurotransmitter or neuropeptide at a time and which have demonstrated, for example, the occurrence in amphioxus of neurons containing cholecystokinin, oxytocin, vasopressin, luteinizing hormone and FMRFamide [31,32]. More recently, serotonergic, dopaminergic and cholinergic neurons have also been reported in *B. floridae* embryos and larvae [21-24], and the distribution of dopamine, serotonin and GABA has been described in amphioxus adults [33,34]. Moreover, by using HPLC, glutamate, aspartate, glycine, alanine, serine and GABA have been identified in the nervous system of the amphioxus *B. lanceolatum *[35]. 

### Interpreting the organization of neuronal cell types in the developing amphioxus nervous system

Previous ultrastructural studies on the architecture and cellular organization of the larval amphioxus CNS have provided a detailed morphological map of different neuronal types [37-39]. The majority of these transmission electron microscope (TEM)-based neuroanatomical reconstructions in amphioxus have been carried out on 8-day and 12.5-day larvae and thus on developmental stages older than the material used in the present study. Comparisons of these morphological data with our neurochemical map will hence need to take these stage differences into account.

On the basis of morphological and topological features, three types of motoneurons have been identified in the ventrolateral nerve cord of amphious larvae: (i) dorsal compartment motoneurons (DC) innervating the dorsal fast fibers of the myotome, (ii) ventral compartment motoneurons (VC) innervating the ventral slow fibers of the myotome, (iii) visceral motoneurons (VM) innervating all of the remaining body musculature (Figure 12). The DC motoneurons are confined to the most anterior somites and are generally organized as pairs of cells. According to Lacalli and Kelly [37] at least one pair of DC motoneurons is slightly offset and is located at the level of the center of somite 2, whereas all the other pairs (at least five) are positioned at the junction of the somites. Expression of amphioxus *islet* and *ERR* genes coincides with prospective DC motoneurons [19,20]. VC motoneurons have no evident periodical arrangement along the nerve cord, although a cluster of five VC neurons is found at the level of the posterior end of somite 1 [37,39]. Finally, VM motoneurons seem to be very scarce in young larvae, but at least two have been described located posterior to the junction of somites 2 and 3 [37,39].

Additionaly, different interneurons have been reported in the amphioxus ventrolateral nerve cord [37,39]. At the ultrastructure level, four types of interneurons, described as large paired neurons (LPN1-4), are present in the amphioxus CNS (Figure 12). The first three at the level of the primary motor center (PMC) and the fourth at the level of the junction of somites 3 and 4 [37,39]. Moreover, four ipsilateral projection interneurons (IPNs) have also been described, located predominantly in the most rostral part of the hindbrain posterior to the first pair of DC motoneurons [37,39]. No information is available on the presence of other kinds of interneurons in more posterior regions of the amphioxus nerve cord.

In a previous report on the expression of the CGL (for cholinergic gene locus, i.e. ChAT/VAChT) [22], we have tentatively assigned a cholinergic neurotransmission to some of the ventrolateral neurons identified by TEM, most of which are motoneurons. This correlation of cell types identified by TEM and neurons expressing specific sets of neurotransmitters can now be expanded. We show, for instance, that cholinergic neurons are generally organized as discontinuous rows of cells starting anteriorly at the PMC and stretching posteriorly to the junction of somites 6 and 7 (Figure 3). Some of the most anterior cholinergic cells, located in proximity of the posterior end of somite 1, are probably VC motoneurons, whereas the pairs of cholinergic neurons at the junctions of the somites (from somites 2 and 3 to somites 5 and 6) are likely DC motoneurons, plus an additional pair of neurons located at the level of somite 2 (Figures 3 and 12). Additionally, the cholinergic neurons located just posterior to the junction of somites 2 and 3 might correspond to VM motoneurons (Figures 3 and 12).

One subset of cholinergic neurons identified in the CNS of developing amphioxus are most likely interneurons [22], which would correlate very well with the situation in vertebrates [40]. For example, LPN3 located at the junction of somites 1 and 2 is a potential candidate for such a cholinergic amphioxus interneuron, although LPN3 could alternatively be interpreted as a pair of glutamatergic excitatory neurons located inside the PMC (Figures 12 and 13). In contrast, our data suggest that the anterior LPNs (LPN1 and 2) and IPNs (on the level of somite 2) are GABAergic and GABAergic/glycinergic, respectively (Figures 12 and 13).

In our neurochemical map, some neurons cannot be ascribed to one of the categories of motoneurons and interneurons identified by TEM [37]. This is, for instance, the case for the cluster of cholinergic, glycinergic and glutamatergic neurons located just posterior to the first pigment spot as well as for the aggregation of a number of different neuronal types (expressing *GABA*, *VGLUT* or *ChAT*) at the level of the PMC (Figures 12 and 13). The PMC is a major integrative center of sensory and motor stimuli controlling the early locomotory activities of the larva, which could explain this concentration of different neuronal types. Moreover, the PMC interneurons (GABAergic and glutamatergic) probably connect to rostral sensory cells and project towards the posterior nerve cord, which likely contributes to locomotion and the startle response [41,42].

### GABAergic and glycinergic neurons in vertebrates and invertebrates

GABA is a major inhibitory neurotransmitter in both vertebrates and invertebrates [43-46]. In comparison to other animals, the distribution of GABA in the CNS of amphioxus embryos and larvae seems to be more discrete. In the CNS of the ascidian *Ciona intestinalis*, GABA is widely expressed and is associated with neural regions responsible for processing sensory information and motor integration, such as the sensory vesicle and the anterior visceral ganglion (Figure 13) [47]. Interestingly, while a majority of neurons of the sensory vesicle are GABAergic, only very few cells of the anterior visceral ganglion contain GABA [47]. In the *C. intestinalis* peripheral nervous system (PNS), there are only very few GABAergic cells, the majority of which are associated with the palps (Figure 13).

Unlike GABAergic cells, which are widespread in the vertebrate brain, most glycinergic neurons in the vertebrate CNS are restricted to the rhombencephalon and the spinal cord. For instance, in embryos of the frog *Xenopus laevis* the first glycinergic cells appear in the caudal hindbrain region and subsequently extend to the spinal cord [48], which was also observed in lamprey embryos [49,50]. In *C. intestinalis* larvae, two pairs of glycinergic neurons have been reported in the tail [51] (Figure 13). These cells, called ACINs (for anterior caudal inhibitory neurons), are located in the anterior nerve cord, just posterior to the cholinergic motoneurons of the visceral ganglion, and act as a component of a neural circuit controlling alternative muscle contraction of the larva.

The distribution of glycinergic neurons in amphioxus is quite similar to that observed in the vertebrate CNS. Two pairs of glycinergic cells are visible in the hindbrain region at the neurula stage. At subsequent stages of development, two further clusters, intermingled with GABA cells, appear more anteriorly at the level of somites 2 and 3 (Figure 13). The present results thus reveal a dynamic developmental pattern of the glycinergic system in amphioxus, including early and late glycinergic neuron populations that might correlate with maturation changes occurring during brain differentiation.

### Comparison of glutamatergic neurons in vertebrates and invertebrates

Glutamate is the predominant excitatory neurotransmitter in the CNS of invertebrates and vertebrates [52,53]. Glutamate is also one of the major neurotransmitters used in the vertebrate retina pathway, with the three VGLUTs being differentially expressed in specific classes of retina neurons [54]. Glutamate is further known to be present in the vestibular hair cells of different vertebrates [55-57] as well as in mechanosensory neurons of invertebrates [58]. In *C. intestinalis*, one *VGLUT* gene has been identified, which is expressed in both the central and the peripheral nervous system (Figure 13) [58]. More specifically, *VGLUT* is found in sensory organs (photoreceptor cells of ocellus and otolith) and interneurons of the posterior sensory vesicle. Moreover, most of the peripheral sensory neurons found in the larval head and tail are also glutamatergic.

In amphioxus, *VGLUT* is expressed at the neurula stage in a few cells of the nerve cord and in subpopulations of ectodermal cells. These ectodermal cells correspond to a subgroup of primary sensory cells expressing neural markers, such as *Hu/Elav*, *ERR*, *β-tubulin*, *Delta*, *Brn3 (POU-IV)* and *synapsin*[20,59-63]. In the amphioxus CNS, glutamatergic neurons are detected in the most anterior and the central region of the cerebral vesicle (Figure 13). Moreover, whereas most of the amphioxus hindbrain is devoid of glutamatergic cells at least until 36-hr, a cluster of *VGLUT* cells is present in the PMC. The expression of *VGLUT* in some cells of the frontal eye complex of amphioxus suggests that, comparable to vertebrate retina cells, some of the photoreceptor cells and/or neurons in amphioxus are also glutamatergic. The posterior end of the amphioxus hindbrain, caudal to the first pigment spot, contains clusters of ventrolateral and dorsolateral *VGLUT* cells (Figure 13). The dorsolateral cluster has previously been homologized with Rohon Beard sensory neurons expressing the neural marker *islet*[19,37]. Since several anamniote vertebrates exhibit Rohon Beard sensory neurons with a glutamatergic phenotype [64,65], our data lend further support to this homology and suggest that glutamatergic transmission is likely to be a common feature of at least certain chordate sense organs.

### The serotonergic and dopaminergic systems

Serotonin (5-HT) is one of the most widespread signaling molecules of metazoans [66-68] and probably even of single-celled eukaryotes, where it can modulate swimming behavior and growth [69]. Dopamine (DA), a neurochemical molecule belonging to the catecholamines, has been identified in most metazoan phyla and hence represents another ancient neurotransmitter [70].

In tetrapod vertebrates, 5-HT neurons are located mainly in the raphe region of the hindbrain, whereas DA-synthesizing nuclei are located within the forebrain and the midbrain [71]. However, in teleosts, for example, 5-HT neurons are also conspicuous in the hypothalamus, where serotonin can modulate neurohormone secretion [72-75]. It is well known that Shh and FGF8 signaling provide essential information to specify the fates and initial positions of DA and 5-HT neurons in the ventral midbrain and rostral hindbrain of vertebrates. These signaling cascades also seem to be important for the expression of early neural patterning genes, such as *Pax2*, *Pax5*, *Wnt1* and *Engrailed*, which establish a fundamental organizing center at the boundary of the midbrain and the hindbrain [76]. This midbrain-hindbrain boundary (MHB) acts as organizing center to direct the patterning of the adjacent neural territories and of the localization and size of the DA and 5-HT cell populations [71].

In the *C. intestinalis* larva, the *TpH* gene is expressed in cells of the visceral ganglion [77], whereas the *TH* gene (that encodes the tyrosine hydroxylase enzyme involved in DA synthesis) is expressed in the coronet cells of the ventral sensory vesicle, a region possibly homologous to the vertebrate hypothalamus (Figure 13) [33]. In amphioxus, 5-HT neurons are mainly located in the cerebral vesicle, whereas TH-labeled cells were described in the lamellar organ and adjacent ventrolateral cells (Figure 13) [21]. Taken together, since in amphioxus the first serotonergic and dopaminergic populations appear very close together in the cerebral vesicle, the ontogeny of the serotonergic and dopaminergic systems in amphioxus seem to differ substantially from the ontogeny of these systems in vertebrates. This difference between amphioxus and vertebrates might not be surprising given the absence in amphioxus of a vertebrate-like MHB organizer, which, in vertebrates, is required for defining the size and localization of the DA and 5-HT cell populations [78,79].

## Conclusions

Our neurochemical map of the amphioxus CNS reveals that the developing amphioxus nervous system is characterized by a strict regionalization and segmented organization of discrete groups of neuronal cell types. At the neurula stage, GABAergic/glycinergic as well as cholinergic neurons show a segmented distribution in the hindbrain, while glutamatergic, serotonergic and dopaminergic neurons are detectable in very restricted groups of neurons with precise locations along the anteroposterior axis of the CNS.

## Methods

### Animal collection and RNA preparation

Amphioxus adults (*B. floridae*) were collected in Tampa Bay, Florida, USA, and electrostimulated to induce gamete release. Eggs were fertilized, and embryos were cultured and fixed according to the published methods [80]. Embryos were collected at appropriate stages by low speed centrifugation and were frozen for RNA extraction or fixed for whole mount in situ hybridization. Following RNA extraction, the RNA was treated with RNAse-free DNAse I (Ambion Europe, UK) according to the manufacturer's recommendations to remove contaminating genomic DNA. First-strand cDNA was synthesized with 5 μg of RNA using the SuperScript first-strand synthesis system (Invitrogen, USA) and oligo(dT) primers.

### Gene cloning and sequence analyses

The *B. floridae* genome sequence [81] was screened with vertebrate and invertebrate VGAT, GAD, SERT, TpH and VGLUT sequences using the TBLASTN algorithm [82] to identify candidate gene fragments. The automated gene annotation of the retained amphioxus sequences was verified and expanded by protein predictions obtained with the GenScan [83] and GenomeScan [84] programs. The resulting amino acid sequences of the amphioxus VGAT, GAD, SERT, TpH and VGLUT candidates were aligned with different vertebrate and invertebrate VGAT, GAD, SERT, TpH and VGLUT sequences using Clustal W v.1.83 [85] and subjected to phylogenetic tree reconstruction analyses. The phylogenetic trees were reconstructed using the Neighbor Joining (NJ) and Maximum Likelihood (ML) methods with distance estimated by JTT amino acid matrix implemented in the program MEGA 5 [86]. Robustness of the resulting trees was calculated by bootstrap analyses in 1,000 replicates. The sequences used for calculating the phylogenies are provided in Additional file 1 and the resulting trees are presented in Additional file 2.

In sum, the *in silico* analyses yielded single amphioxus VGAT, GAD, SERT, TpH and VGLUT sequences that were used to design specific primers for PCR amplification. The following primers were used to amplify partial sequences of: VGAT, 5' primer GGTCCAGTGTTTGTACGAGGA and 3' primer GATCCCACTTCAGCTTCATGT; GAD, 5' primer ACATCCCCGCTTTTTCAAC and 3' primer GGAGATAGAACGGCTGGGA; SERT, 5' primer TGCGTTCCTTGTCCCTTATTTCA and 3' primer CCCGCGGGTACTCGTCACTCAG; TpH 5’ primer AAGCCGACAAGACCCGAATGAAC and 3’ primer TCTAAGGCGTGGCTAATGGTGTCC; VGLUT 5’ primer TGGGGATACATCGTCACTCA and 3’ primer GGGAGCAATGTCAAGATGGT. An embryonic cDNA library was used to isolate amphioxus amplicons. In some cases, the amplicons were identified by RT-PCR on RNA samples from amphioxus adults. PCR experiments were carried out in a 50 μl reaction mixture using the Hot Master mix according to the manufacturer's instructions (Eppendorf, Italy). The PCR products were directly cloned using a TOPO TA cloning kit (Invitrogen, USA) and subsequently sequenced using a 377 PerkinElmer sequencer (PerkinElmer, USA).

### In situ hybridization and histological analysis

The cDNA sequences corresponding to the clones of amphioxus *VGAT, GAD, SERT, TpH and VGLUT* isolated by PCR were used as templates for in vitro transcription using a DIG RNA labeling kit according to the supplier's instructions (Roche, Italy). Cholinergic neurons were studied using a riboprobe against VAChT previously isolated in the laboratory [22]. In situ hybridization analysis on amphioxus embryos was carried out following the published protocol [80]. Labeled whole mount embryos were photographed using an Olympus IX71 microscope (Olympus, Italy), and subsequently counterstained with 1% Ponceau S in 1% acetic acid, dehydrated in ethanol, embedded in Spurr's resin and serially sectioned at 3–4 μm. Negative control experiments were performed using sense riboprobes and no specific signal was obtained.

For double in situ hybridization assays, embryos were hybridized simultaneously with two probes labeled with fluorescein and digoxigenin and developed with the appropriate antibody conjugated to alkaline phosphatase (Boehringer, Germany). Double-staining in situ hybridization was performed as previously described [22].

Confocal images (1024 X 1024 X 8 bit) were acquired on a Leica TCS SP5 AOBS confocal laser scanning microscope (Leica Microsystems, Germany). The NBT/BCIP signal was acquired with a pinhole of 2 airy units and a 633 nm gas laser with the detection window set to 630–640 nm. The red fluorescence of Fast Red (Ex Max: 553 nm, Em Max: 619 nm) was excited at 514 nm and collected with a 590 nm longpass filter. Serial optical sections were taken at a z-step of 0.5 mm. The Leica Confocal Software program was used for image acquisition, storage and analysis (Leica Microsystems, Germany).

## Authors’ contributions

SC conceived the study. SC and LM identified amphioxus sequences, reconstructed molecular phylogenies and carried out in situ hybridization assays. SC and PR obtained the confocal images. SC, MP and MS drafted and wrote the manuscript. All authors have read and approved the final version of the manuscript. This work was supported by MIUR (PRIN 20088JEHW3-001) (to SC and MP) and by funds from ANR (ANR-09-BLAN-0262-02 and ANR-11-JSV2-002-01) and CNRS (to MS). All authors read and approved the final manuscript.

## Supplementary Material

Additional file 1 **FASTA sequences of VGLUT, TpH, SERT, GAD and VGAT proteins used for the construction of the phylogenetic trees shown in Additional file**2**.** Click here for file

Additional file 2 **Rooted phylogenetic trees of VGLUT, TpH, SERT, GAD and VGAT proteins. **The results of both Maximum Likelihood (ML) and Neighbor Joining (NJ) analyses are shown. In each tree, the robustness of the phylogenetic branching patterns was assessed by bootstrap analyses in 1,000 resampling replicates. Taxonomic abbreviations are as follows: human (*Homo sapiens*), pig (*Sus scrofa*), sheep (*Ovis aries*), rat (*Rattus norvegicus*), mouse (*Mus musculus*), chicken (*Gallus gallus*), African clawed frog (*Xenopus laevis*), zebrafish (*Danio rerio*), amphioxus (*Branchiostoma floridae*), sea squirt (*Ciona intestinalis*), sea urchin (*Strongylocentrotus purpuratus*), sea slug (*Aplysia californica*), fruit fly (*Drosophila melanogaster*), nematode worm (*Caenorhabditis elegans*). Click here for file
