# Supplementary material for: A neurochemical map of the developing amphioxus nervous system
Source: BMC Neurosci. 2012 Jun 7;13:59. doi: 10.1186/1471-2202-13-59 (PMC3484041; doi:10.1186/1471-2202-13-59)
Supplement: Additional file 1 — FASTA sequences of VGLUT, TpH, SERT, GAD and VGAT proteins used for the construction of the phylogenetic trees shown in Additional file2. [file 1471-2202-13-59-S1.pdf]

Vesicular Glutamate Transporter (VGLUT) proteins

>zebrafish_VGLUT1 ENSDART00000104481
MEIRPDRFKAQAGKTLGKIHRLLEKRQQNGETIELSAEGRPELVEEKELPIVDCTCFGLPRRYIIAILSG
LGFCISFGIRCNLGVAIVSMVNDHTVYSGKKPVIVAAQFTWDPETVGMIHGSFFWGYIVTQIPGGFICQK
FAANRVFGFAIVATSTLNMLIPSAARVHYGCVIMVRVCQGLVEGVSYPACHGIWAKWAPPLERSRLATTA
FCGSYAGAVVAMPLAGVLVQYTGWSSVFYVYGSVGIFWYLFWILVSYESPAAHPTITPEERKYIEDAIGE
SAGLVNPLQKFKTPWRHFFTSMPVYAIIVANFCRSWTFYLLLISQPAYFEEVFGFEISKVGMVSALPHLV
MTIIVPIGGQLADYLRTHNLMTTTNVRKLMNCGGFGMEATLLLVVGFSHTKGVAISFLVLAVGFSGFAIS
GFNVNHLDIAPRYASILMGISNGVGTLSGMVCPLIVGAMTKHKTREEWQYVFLIAALVHYGGVIFYGLFA
SGEKQPWADAENTSEEKCGILGEDELANETEELYRTGGQYGAINNPHVGGPNGGGAGAGAGWVSDWDKTE
EYVQPVGTNSYLYGGEGERELT
>human_VGLUT1 NP_064705.1
MEFRQEEFRKLAGRALGKLHRLLEKRQEGAETLELSADGRPVTTQTRDPPVVDCTCFGLPRRYIIAIMSG
LGFCISFGIRCNLGVAIVSMVNNSTTHRGGHVVVQKAQFSWDPETVGLIHGSFFWGYIVTQIPGGFICQK
FAANRVFGFAIVATSTLNMLIPSAARVHYGCVIFVRILQGLVEGVTYPACHGIWSKWAPPLERSRLATTA
FCGSYAGAVVAMPLAGVLVQYSGWSSVFYVYGSFGIFWYLFWLLVSYESPALHPSISEEERKYIEDAIGE
SAKLMNPLTKFSTPWRRFFTSMPVYAIIVANFCRSWTFYLLLISQPAYFEEVFGFEISKVGLVSALPHLV
MTIIVPIGGQIADFLRSRRIMSTTNVRKLMNCGGFGMEATLLLVVGYSHSKGVAISFLVLAVGFSGFAIS
GFNVNHLDIAPRYASILMGISNGVGTLSGMVCPIIVGAMTKHKTREEWQYVFLIASLVHYGGVIFYGVFA
SGEKQPWAEPEEMSEEKCGFVGHDQLAGSDDSEMEDEAEPPGAPPAPPPSYGATHSTFQPPRPPPPVRDY
>human_VGLUT2 NP_065079.1
MESVKQRILAPGKEGLKNFAGKSLGQIYRVLEKKQDTGETIELTEDGKPLEVPERKAPLCDCTCFGLPRR
YIIAIMSGLGFCISFGIRCNLGVAIVDMVNNSTIHRGGKVIKEKAKFNWDPETVGMIHGSFFWGYIITQI
PGGYIASRLAANRVFGAAILLTSTLNMLIPSAARVHYGCVIFVRILQGLVEGVTYPACHGIWSKWAPPLE
RSRLATTSFCGSYAGAVIAMPLAGILVQYTGWSSVFYVYGSFGMVWYMFWLLVSYESPAKHPTITDEERR
YIEESIGESANLLGAMEKFKTPWRKFFTSMPVYAIIVANFCRSWTFYLLLISQPAYFEEVFGFEISKVGM
LSAVPHLVMTIIVPIGGQIADFLRSKQILSTTTVRKIMNCGGFGMEATLLLVVGYSHTRGVAISFLVLAV
GFSGFAISGFNVNHLDIAPRYASILMGISNGVGTLSGMVCPIIVGAMTKNKSREEWQYVFLIAALVHYGG
VIFYAIFASGEKQPWADPEETSEEKCGFIHEDELDEETGDITQNYINYGTTKSYGATTQANGGWPSGWEK
KEEFVQGEVQDSHSYKDRVDYS
>human_VGLUT3 NP_647480.1
MPFKAFDTFKEKILKPGKEGVKNAVGDSLGILQRKIDGTTEEEDNIELNEEGRPVQTSRPSPPLCDCHCC
GLPKRYIIAIMSGLGFCISFGIRCNLGVAIVEMVNNSTVYVDGKPEIQTAQFNWDPETVGLIHGSFFWGY
IMTQIPGGFISNKFAANRVFGAAIFLTSTLNMFIPSAARVHYGCVMCVRILQGLVEGVTYPACHGMWSKW
APPLERSRLATTSFCGSYAGAVVAMPLAGVLVQYIGWSSVFYIYGMFGIIWYMFWLLQAYECPAAHPTIS
NEEKTYIETSIGEGANVVSLSKFSTPWKRFFTSLPVYAIIVANFCRSWTFYLLLISQPAYFEEVFGFAIS
KVGLLSAVPHMVMTIVVPIGGQLADYLRSRQILTTTAVRKIMNCGGFGMEATLLLVVGFSHTKGVAISFL
VLAVGFSGFAISGFNVNHLDIAPRYASILMGISNGVGTLSGMVCPLIVGAMTRHKTREEWQNVFLIAALV
HYSGVIFYGVFASGEKQEWADPENLSEEKCGIIDQDELAEEIELNHESFASPKKKMSYGATSQNCEVQKK
EWKGQRGATLDEEELTSYQNEERNFSTIS
>mouse_VGLUT1 NP_892038.2
MEFRQEEFRKLAGRALGRLHRLLEKRQEGAETLELSADGRPVTTHTRDPPVVDCTCFGLPRRYIIAIMSG
LGFCISFGIRCNLGVAIVSMVNNSTTHRGGHVVVQKAQFNWDPETVGLIHGSFFWGYIVTQIPGGFICQK
FAANRVFGFAIVATSTLNMLIPSAARVHYGCVIFVRILQGLVEGVTYPACHGIWSKWAPPLERSRLATTA
FCGSYAGAVVAMPLAGVLVQYSGWSSVFYVYGSFGIFWYLFWLLVSYESPALHPSISEEERKYIEDAIGE
SAKLMNPVTKFNTPWRRFFTSMPVYAIIVANFCRSWTFYLLLISQPAYFEEVFGFEISKVGLVSALPHLV
MTIIVPIGGQIADFLRSRHIMSTTNVRKLMNCGGFGMEATLLLVVGYSHSKGVAISFLVLAVGFSGFAIS
GFNVNHLDIAPRYASILMGISNGVGTLSGMVCPIIVGAMTKHKTREEWQYVFLIASLVHYGGVIFYGVFA
SGEKQPWAEPEEMSEEKCGFVGHDQLAGSDESEMEDEAEPPGAPPAPPPSYGATHSTVQPPRPPPPVRDY
>mouse_VGLUT3 NP_892004.1
MPFKAFDTFKEKILKPGKEGVKNAVGDSLGILQRKIDGTNEEEDAIELNEEGRPVQTSRAHRPVCDCSCC
GIPKRYICDCSCCGIPKRYIIAVMSGLGFCISFGIRCNLGVAIVEMVNNSTVYVDGKPEIQTAQFNWDPE
TVGLIHGSFFWGYIVTQIPGGFISNKFAASRVFGAAIFLTSTLNMFIPSAARVHYGCVMGVRILQGLVEG
VTYPACHGMWSKWAPPLERSRLATTSFCGSYAGAVVAMPLAGVLVQYIGWASVFYIYGMFGIIWYMFWLL
QAYECPAAHPTISNAERTYIETSIGEGANLASLSKFNTPWRRFFTSLPVYAIIVANFCRSWTFYLLLISQ
PAYFEEVFGFAISKVGLLSAVPHMVMTIVVPIGGQLADYLRSRKILTTTAVRKIMNCGGFGMEATLLLVV
GFSHTKGVAISFLVLAVGFSGFAISGFNVNHLDIAPRYASILMGISNGVGTLSGMVCPLIVGAMTKHKTR
EEWQNVFLIAALVHYSGVIFYGVFASGEKQDWADPENLSEDKCGIIDQDELAEETELNHETFVSPRKKMS
YGATTQNCEVQKTEWRQQRESAFDGEEPLSYQAEGDFSETS
>zebrafish_VGLUT2.1 NP_001122293.1

METPREPAGFSKEGLKQLAGKTLGHVYRVIEKRQKPGENIELTEDGRPAQINERKAPLCDCTCFGLPRRY
IIAIMSGLGFCISFGIRCNLGVAIVSMVNNSTIHLNGKIIIKEKAKFNWDPETVGLIHGSFFWGYIVTQI
PGGYISSRLAANRVFGAAILLTSTLNMFIPSAARGHYGCVIFVRILQGLVEGVTYPACHGIWSKWAPPLE
RSRLATTSFCGSYAGAVIAMPLAGILVQYTGWSSVFYVYGCFGIFWYMFWILVSYESPAEHPTITAEERC
YIEESIGESAKLLGPADKFKTPWRKFFTSMPVYAIIVANFCRSWTFYLLLISQPAYFEEVFGFEISKVGM
LSALPHLVMTIIVPIGGQLADHLRSKNILSTTTVRKIMNCGGFGMEATLLLIVGYSHSKGVAISFLVLAV
GFSGFAISGFNVNHLDIAPRYASILMGISNGVGTLSGMVCPLIVGAMTKHKTREEWQYVFLIASLVHYGG
VIFYGIFASGEKQPWADPELTSDEKCGFIDEDELAEETGDITQSYGALGAPAKSYGATTQLNGGWAEGWD
KREEYVQDGVEEGGYGYRQGGNYS
>zebrafish_VGLUT2.2 NP_001009982.1
MDTVKERVLAPGKEKMRNLAGKTLGHMHRVMERKQKTGEVIELTEDGRPMHMPEKKAPLVDCTCFGLPRR
YIIAIMSGLGFCISFGIRCNLGVAIVDMVNNSTIHKGGKIIIKGKAKFNWDPETVGMIHGSFFWGYTVTQ
IPGGYISSRLAANRVFGAAILLTSTLNMFIPSAARVHYGCVMFVRILQGLVEGVTYPACHGIWSKWAPPL
ERSRLATTSFCGSYAGAVVAMPLAGILVQYSGWSSVFYIYGSFGIVWYMFWILVSYESPADHPTITDEER
TYIEESIGESAKLLGAMEKYKTPWRKFFTSMPVYAIIVANFCRSWTFYLLLISQPAYFEEVFGFEISKVG
MVSALPHLVMTIIVPIGGQLADYLRSKNILTTTTVRKIMNCGGFGMEATLLLVVGFSHSKGVAISFLVLA
VGFSGFAISGFNVNHLDIAPRYASILMGISNGVGTLSGMVCPLIVGAMTKNKTREEWQNVFLIASLVHYG
GVIFYGIFASGEKQPWADPEETSDEKCGFIDEDELAEETGDITLSHAPFGAQGALGAPAKTYGATTQLNG
GWAKGWEKTEEFIQEDAERTYTGDGYS
>zebrafish_VGLUT3 NP_001076304.1
MPLGGFAGLKEKLNPGKEELKNNVGDSLGNLQKKIDGSNVTEEDNIELTEDGRPVAAPKRSPPLLDCGCF
GLPKRYIIAMLSGLGFCISFGIRCNLGVAIVEMVNNNTVYINGTAVMQPAQFNWDPETVGLIHGSFFWGY
IVTQIPGGFISNKLAANRVFGAAIFLTSVLNMFIPSAARVHYGCVMFVRILQGLVEGVTYPACHGMWSKW
APPLERSRLATTSFCGSYAGAVIAMPLAGILVQYVGWPSVFYIYGVFGIIWYIFWILLAYNSPAVHPTIS
EEERNYIETSIGEGANLMSSTEKFKTPWREFFTSMPVYAIIVANFCRSWTFYLLLISQPAYFEEVFGFPI
SKVGILSAVPHMVMTIIVPIGGQLADFLRSRKILSTTTVRKIMNCGGFGMEATLLLVVGFSHTRAVAISF
LILAVGFSGFAISGFNVNHLDIAPRYASILMGISNGVGTLSGMVCPLIVGALTKHKTRLEWQHVFVIASM
VHYTGVIFYAIFASGEKQDWADPENTSDEKCGIIGEDELADETEPSSDSGLATRQKTYGTTDNSSGRKQG
WKKKRGVTMQAEDDHESNHYENGEYQTQYQ
>rattus_VGLUT1 NP_446311.1
MEFRQEEFRKLAGRALGRLHRLLEKRQEGAETLELSADGRPVTTHTRDPPVVDCTCFGLPRRYIIAIMSG
LGFCISFGIRCNLGVAIVSMVNNSTTHRGGHVVVQKAQFNWDPETVGLIHGSFFWGYIVTQIPGGFICQK
FAANRVFGFAIVATSTLNMLIPSAARVHYGCVIFVRILQGLVEGVTYPACHGIWSKWAPPLERSRLATTA
FCGSYAGAVVAMPLAGVLVQYSGWSSVFYVYGSFGIFWYLFWLLVSYESPALHPSISEEERKYIEDAIGE
SAKLMNPVTKFNTPWRRFFTSMPVYAIIVANFCRSWTFYLLLISQPAYFEEVFGFEISKVGLVSALPHLV
MTIIVPIGGQIADFLRSRHIMSTTNVRKLMNCGGFGMEATLLLVVGYSHSKGVAISFLVLAVGFSGFAIS
GFNVNHLDIAPRYASILMGISNGVGTLSGMVCPIIVGAMTKHKTREEWQYVFLIASLVHYGGVIFYGVFA
SGEKQPWAEPEEMSEEKCGFVGHDQLAGSDESEMEDEVEPPGAPPAPPPSYGATHSTVQPPRPPPPVRDY
>rattus_VGLUT2 NP_445879.1
MESVKQRILAPGKEGIKNFAGKSLGQIYRVLEKKQDNRETIELTEDGKPLEVPEKKAPLCDCTCFGLPRR
YIIAIMSGLGFCISFGIRCNLGVAIVDMVNNSTIHRGGKVIKEKAKFNWDPETVGMIHGSFFWGYIITQI
PGGYIASRLAANRVFGAAILLTSTLNMLIPSAARVHYGCVIFVRILQGLVEGVTYPACHGIWSKWAPPLE
RSRLATTSFCGSYAGAVIAMPLAGILVQYTGWSSVFYVYGSFGMVWYMFWLLVSYESPAKHPTITDEERR
YIEESIGESANLLGAMEKFKTPWRKFFTSMPVYAIIVANFCRSWTFYLLLISQPAYFEEVFGFEISKVGM
LSAVPHLVMTIIVPIGGQIADFLRSKQILSTTTVRKIMNCGGFGMEATLLLVVGYSHTRGVAISFLVLAV
GFSGFAISGFNVNHLDIAPRYASILMGISNGVGTLSGMVCPIIVGAMTKNKSREEWQYVFLIAALVHYGG
VIFYALFASGEKQPWADPEETSEEKCGFIHEDELDEETGDITQNYINYGTTKSYGATSQENGGWPNGWEK
KEEFVQESAQDAYSYKDRDDYS
>rattus_VGLUT3 NP_714947.1
MPFNAFDTFKEKILKPGKEGVKNAVGDSLGILQRKLDGTNEEGDAIELSEEGRPVQTSRARAPVCDCSCC
GIPKRYIIAVMSGLGFCISFGIRCNLGVAIVEMVNNSTVYVDGKPEIQTAQFNWDPETVGLIHGSFFWGY
IVTQIPGGFISNKFAANRVFGAAIFLTSTLNMFIPSAARVHYGCVMCVRILQGLVEGVTYPACHGMWSKW
APPLERSRLATTSFCGSYAGAVVAMPLAGVLVQYIGWASVFYIYGMFGIIWYMFWLLQAYECPAVHPTIS
NEERTYIETSIGEGANLASLSKFNTPWRRFFTSLPVYAIIVANFCRSWTFYLLLISQPAYFEEVFGFAIS
KVGLLSAVPHMVMTIVVPIGGQLADYLRSRKILTTTAVRKIMNCGGFGMEATLLLVVGFSHTKGVAISFL
VLAVGFSGFAISGFNVNHLDIAPRYASILMGISNGVGTLSGMVCPLIVGAMTKHKTREEWQNVFLIAALV
HYSGVIFYGVFASGEKQDWADPENLSEEKCGIIDQDELAEETELNHEAFVSPRKKMSYGATTQNCEVQKT
DRRQQRESAFEGEEPLSYQNEEDFSETS
> nematode worm_VGLUT AAC64972.1
MVGEPLAKMTAAAASATGAAPPQQMQEEGNENPMQMHSNKVLQVMEQTWIGKCRKRWLLAILANMGFMIS
FGIRCNFGAAKTHMYKNYTDPYGKVHMHEFNWTIDELSVMESSYFYGYLVTQIPAGFLAAKFPPNKLFGF

GIGVGAFLNILLPYGFKVKSDYLVAFIQITQGLVQGVCYPAMHGVWRYWAPPMERSKLATTAFTGSYAGA
VLGLPLSAFLVSYVSWAAPFYLYGVCGVIWAILWFCVTFEKPAFHPTISQEEKIFIEDAIGHVSNTHPTI
RSIPWKAIVTSKPVWAIIVANFARSWTFYLLLQNQLTYMKEALGMKIADSGLLAAIPHLVMGCVVLMGGQ
LADYLRSNKILSTTAVRKIFNCGGFGGEAAFMLIVAYTTSDTTAIMALIAAVGMSGFAISGFNVNHLDIA
PRYAAILMGFSNGIGTLAGLTCPFVTEAFTAHSKHGWTSVFLLASLIHFTGVTFYAVYASGELQEWAEPK
EEEEWSNKELVNKTGINGTGYGAAETTFTQLPAGVDSSYQAQAAPAPGTNPFASAWDEHGSSGVVENPHY
QQW
>sea squirt_VGLUT NP_001122357.1
MSNNKADVGFATASHDLLRTIQNGFRSVLYRFTGIGNPPYQEQTLDRNSTLNEISRNSQEHGEDHFEEDV
PQTNNKWMWYAMCLWLPKRYMMAFLSGFGFCITFGMRCNLGVAMVEMANNYTETLENGTKVIMPPDLSWS
SEKQGFIHGSFFWGYIITQVPGGYLASRLRANRVFGVAILCTCLLNMFLPAAAKAHWVVFVIVRVMQGLA
EGVLYPSCHGIWSKWAPPLERSRLATISFSGSYAGAVIGMPIGGMLVEYAGWPSVFYVFGSCGIAWFFLW
TFTSYDSPASHPHIRRSERIYIEESIGKSDCATIPMVRTLFNTLGTPWKKFLTSLPVWAIIVANFCRSWT
FYLLIISQPAYFEQVLKYDISQLGFLAAVPHLVMTIIVPFGGVLADFLRKKEILSTTNVRKVMNCGGFGM
EALFLLILACSHGHTASVVCLVFAVGFSGFAISGFNVNHLDIAPRYASILMGLSNGAGTLSGMICPLLVS
YITRTKTEEDWKVVFVIASCIHFSGVIFYAFFASGERQPWADPPQEEVGILDGENFAASPSMTSFRRRSG
MSRGSSSSDSMFGEGPDDLYVKKLTNGVYNPSFEGGPRSKRFSTAGEEEDQQMTEAQMKRTMYVTSKLTS
DGPPYDVVTETVQQPAVDELFQGVREDEKYYKH
>fruit fly_VGLUT NP_608681.2
MKGLTAFKEKATGVFGGLKPNMEKFEISQSYHGGHGGYEEMEGGDREGRGPGGGHAYDDDDDRPDSPASF
EEIERPPLRKIDKYCKAECPCMPARYTIATMACVGFMIAFGMRCNMSAAKLKGEHNGTVFMNWTVAVESH
VDSSFFWGYLVTQIPGGFIASKFPANKIFGLSIVSSATLHLFVPFAMTLMHGHVVICVRVLQGLFEGVTY
PACHGIWRFWAPPMERSRLATLAFSGSYAGVVVGLPLSGLLADAVGYQAPFYAYGVFGIIWYMFWIWLCF
ENPRKHPAISIPELKYIEKSLGESAHPTMPSLKTTPWREMMRSMPVYAIIVANFCRSWNFYLLVLFQSSF
LKHKFGFKVEEAGFVGSLPHLIMTTIVPFGGMLADHLRKNGILSTTNVRKLFNCGGFGMEGLFFLFVAHS
STATGAMFALTCGVAFSGFAISGYNVNHLDIAPRYASILMGLSNGIGTLAGIIVPYALDGLIQANPTGCW
TTVFTLAACVHLVGCTFYGIFASGELQPWAEPPAEEQKVWAPPPGAITNTDPSQAGMLGDYMKETSFGAP
EYTEQSQMQQSTAISYGATGHVANNPFAMASGAPPIAEEDAPPTYGDVTNPGQYGYTQGQMPSYDPQGYQ
QQ
>amphioxus_VGLUT
MFITNIQLSDRFRAFKKDTDEKGLVTHDEAQAQHEEDHPELAALPPGEDEKRPGLCDRTPCANLTKRYQI
AILSCIGFIISFGIRCNMGVAVVDMTNNVTVHVGEGEKPHIQRAEFHWTPEAIGIIHGSFFWGYIVTQIP
GGYLATRFPANRIFGLAILSTASLNMLIPAAAKVHYGCVIAVRILQGLVEGVTYPAQHGIWSKWAPPLER
SRLVTMSFCGSYAGAVVSMPLSGLTDYAGWPCPFYVYGAAGMIWFIAWMLIAYESPAAHPTITQAERIYI
EDSIGGSTKRTLDMKTPWAKFFTSMPVYAIIVANFCRSWTFYLLLISQPSYFEEVFGFDISQVGILSALP
HLVMTCIVPMGGQLADFLRRRRILSTTNVRKIFNCGGFGMEAVFLLICGYSSRNTAVAIVCLTIAVGFSG
FAISGFNVNHLDIAPRWASVLMGISNGVGTLSGMICPIIVSTVTRHKNAREWQTVFLIAASVHFVGVIFY
AFFASGDVQPWNDDIDGLNGEK
>amphioxus_VGLUT-like1
MITFFLRRFLGKPEEKQNLVSKKKKKKTETTQDDQEPRDHNLRDDLLEEEEDGDRHKKPWRCSHTCNCWC
GSTRYVISVMASLGFIISFGMRCNLGVSIIKMLENTTKAEEEDVNGTSSLFDWQPETVGVVSSAFLWGYL
FTQVPGGYLAARYPANRVFGVALFTTCILNLFLPVSARVGWGALAAVRVLQGLAEGMTYPAAHGIWSNWA
PPLERTRLSTITFAGTYFGAVTGMPLSGLLADLMGWESPFYFYGAIGIMWSCGWYFVSSPSPARHTFLSR
EEQLYIEESIGNKGENKIMEGGVPWRAMLTSMPVYAIIVANFCRSWTFYLLLTSQPIYMQQSYVNYDTTR
IGYLSAIPHFLMGLVVVLGGQLADTLRSRGWSTTVVRKLFNCGGFLSEGVFLLIIAAACNGGPGVIALLTV
AITVAGMAISGYSVNHLDVAPRYASILMGLSNCIGTVGGMLSPLTVGWLTAAYGYRGWSYVFLIAGLVHI
NGVIFYFFFGSGEKQPWADPEPEEEGDVVIYTRDQDEDEDEVEFLWDYEPHDFLRLTRRDNFVQLPATDD
YLYGGVNHRDFEY
>amphioxus_VGLUT-like2
MQIFPRNITKAIVGNTQEERRGGDLATASAAQTLLHRVEVQTRILRATVGQHLPRKDPGPYDLNGCIQYP
RFMPKRYIVAVLACIGFMIQYGIRTNLGIAVVDMVNNHTLGDLEVPAPFHWSQLTVGIIHGSFFWGFLLT
KVLGGYLAVRFPANRVFGASVLCSACLHMVVPVAADLHYGVLIFVRILQGLSEGMTFPSSYGVLSKWAPP
LERSRLTSIMVTGQYAGILFGMPLSGVVTEYAGWPYAFYMYGAFGITWSVIWALCVWESPSKDPTVSEEE
CLFIEKSLGEFYQSSNQQSTAQETPWRDILTSPPVYAILITDTAFKWTLYLLLTNSPSYYMQAFDMQVEA
SGLITGMPFLFLALCLPIAGSVGDWMRTKEVMSTTNVRRLFNTVGLSLQALCFLIIGCTNSSVASMTLLS
IGMASAGLTLSSGYNVNALDISPRYASIIMGLSGAIATLSGILCPIVVGTITVDKTAKQWGYVFIISAVI
LIISVVFYFFLASGETQPWSNPKPRSDGADDDDEEDVFVKKSKLPEWAKKPKMPDWFRTAGDGKPSSPEP
QDEAAGKDAGRKATDKLMARSVVDDGSPIDWVDESVNFVQIDPENPYMYNTERDF
>amphioxus_VGLUT-like3
MVGAIVLGAIIYGLLASAEPLQIVVEKPRPKIKLPKFPFPSISAPKVKIPFFPDWFKKREEPEGEAKEDAG
ETGAPMSWYKKVLDGVPKPDINYPTVDWFQLPTQKALCSCMPKRYLIAMLSCIGFLIEYGARTNMGVAAT

QMVNNEDVMNQLFQPSELEWTMLIVGVIHGAFFLGFLLSRPLGGFLVSRYPATIVFVLSIAVSSLVNLFI
PVAATVHFSILVALRVIQGMSEGLTIPASYGIWTFWAPPAERSKLTSITVTGQYIGIVIGMPVSGVAVFN
IDWRFPFWIYGGVGLVWSIIWYIMVYESPEVDKYLDPEELKFIQDSMTSHPGIISPSEPDAKSEASEAST
EASEPAAKTPTSPASSVGPDLPTQVSPQKTPWRHLLTSLPVYAILVCDTCVKWVVYLMLLNAPMYYVQSF
KVNELEAGAFAGLHFFLLMFGLPLVGSLADWVWRTQSMSTTTMRKLFNTMGMFVEGALLLTVGMTSNIYV
STICLSVALFFQSFPLSAGYNVNALDIAPQFASSIMGLSGSLSTVIGMACPVAIGAITMDKTPEEWNIVF
IVSAGLILCAGLLYLLFGSGEVQDWADFGPNPSVSAGDPVPRGNESGLPNLVFPDPETLLDESVHRVQME
DKDPYMYNLGDRDFSYLDDDDF

Tryptophan Hydroxylase (TpH) proteins

> nematode worm_TpH AAD30115.1
MDSLFQMASAMKFQYYSKKAAGKTMSNSVSMSSDNRMEDFKRRFRRSGSLGIPFVPEEDVKQLFTPTRTV
RREASIREGDEEEGVQILTIIVKSSRVSEDISKMIANLPDHTRIKHLETRDSQDGSSKTMDVLLEIELFH
YGKQEAMDLMRLNGLDVHEVSSTIRPTAIKEQYTEPGSDDATTGSEWFPKSIYDLDICAKRVIMYGAGLD
ADHPGFKDTEYRQRRMMFAELALNYKHGEPIPRTEYTSSERKTWGIIYRKLRELHKKHACKQFLDNFELL
ERHCGYSENNIPQLEDICKFLKAKTGFRVRPVAGYLSARDFLAGLAYRVFFCTQYVRHHADPFYTPEPDT
VHELMGHMALFADPDFAQFSQEIGLASLGASEEDLKKLATLYFFSIEFGLSSDDAADSPVKENGSNHERF
KVYGAGLLSSAGELQHAVEGSATIIRFDPDRVVEQECLITTFQSAYFYTRNFEEAQQKLRMFTNNMKRPF
IVRYNPYTESVEVLNNSRSIMLAVNSLRSDINLLAGALHYIL
>amphioxus_TpH
MFVRSCLRITTKMSGRKLLGRRFSDVFPLNNGTCGPLLNKEKSTKQFTTEEVLWILIRSKISRIFQTVGP
GTRRRISSFPCEETFAPVRTPVILSMRKDVGGLAAVLQLFQEEQVSVLHIESRRSLRRKSEVEIYMDCEA
DKTRMNELICRIQRETKMVKVDTPDSLGKDNGQGEEGFEVPWFPRKISELDKTACRVLMYGNDLDADHPG
FKDNVYRERRKQFAEIALNYKYGQPIPRIKYTEEEVNTWGAVYRELTSLYPTHACQQHLNNLPLLRMYCG
YREDNIPQLEDVSAFLKERTGFQLRPVAGYLTPRDFLAGLAFRVFHCTQYIRHSTDPFYTPEPDCCHELL
GHVPMLADPSFAEFSHEIGLASLGASDEEVQKLATCYFFSVEFGLCKEDGKIRAYGAGLLSSAGELKHAL
TQEDKVLPFDPEAVVQQECLITTYQDVYFLFHSFDEAKEQMRSFAKSIKRPFTVRYNPYTQTVEVLNSTR
QVARVIQELRAELDTISHALDKMDV
>sea squirt_TpH ABI51622.1
MADTRRGQLHHVTSLDDVISDTRAKTEKYSSLRTSIRFHLEYNTTPLSMIFALFEDEGVPVLEVDKSSET
HQVTINVELEKLETILTKLKLFPGISQITKENTKENGIWFPKCLADLDGCAKNVLMYGAELDADHPGFKD
EVYRKRRDYFTKLAMDFRHGDKIPRVEYTKIEIETWGKVYKELMELHPTRACAQHLKNLPLLSEFCKCSE
DNVPQLEDISAFLQSRTGFRIRPAAGFLSPRDFLAGLAFRVFNCTQYIRHHSDPYYTPEPDICHEILGHV
PLLADPEFAQFSQEIGLASLGVSDQDTSKLAGCYLYTVEFGLCKEQDGIKAYGAGLLSSISELKHALSSP
EKVRAFDAVTASCQESHVTAFQPVYFLSPSFSQAKHEMRAFAATLERPFVLSFDEETSSVKVFDKLSSIQ
SAVSKMSHDLVVISKALGEMEKRKIEVC
>human_TpH2 EAW97278.1
MQPAMMMFSSKYWARRGFSLDSAVPEEHQLLGSSTLNKPNSGKNDDKGNKGSSKREAATESGKTAVVFSL
KNEVGGLVKALRLFQEKRVNMVHIESRKSRRRSSEVEIFVDCECGKTEFNELIQLLKFQTTIVTLNPPEN
IWTEEEGKELEDVPWFPRKISELDKCSHRVLMYGSELDADHPGFKDNVYRQRRKYFVDVAMGYKYGQPIP
RVEYTEEETKTWGVVFRELSKLYPTHACREYLKNFPLLTKYCGYREDNVPQLEDVSMFLKERSGFTVRPV
AGYLSPRDFLAGLAYRVFHCTQYIRHGSDPLYTPEPDTCHELLGHVPLLADPKFAQFSQEIGLASLGASD
EDVQKLATCYFFTIEFGLCKQEGQLRAYGAGLLSSIGELKHALSDKACVKAFDPKTTCLQECLITTFQEA
YFVSESFEEAKEKMRDFAKSITRPFSVYFNPYTQSIEILKDTRSIENVVQDLRSDLNTVCDALNKMNQYL
GI
>mouse_TpH2 AAI20515.1
MQPAMMMFSSKYWARRGLSLDSAVPEDHQLLGSLTQNKAIKSEDKKSGKEPGKGDTTESSKTAVVFSLKN
EVGGLVKALRLFQEKHVNMLHIESRRSRRRSSEVEIFVDCECGKTEFNELIQLLKFQTTIVTLNPPESIW
TEEEDLEDVPWFPRKISELDRCSHRVLMYGTELDADHPGFKDNVYRQRRKYFVDVAMGYKYGQPIPRVEY
TEEETKTWGVVFRELSKLYPTHACREYLKNLPLLTKYCGYREDNVPQLEDVSMFLKERSGFTVRPVAGYL
SPRDFLAGLAYRVFHCTQYVRHGSDPLYTPEPDTCHELLGHVPLLADPKFAQFSQEIGLASLGASDEDVQ
KLATCYFFTIEFGLCKQEGQLRAYGAGLLSSIGELKHALSDKACVKSFDPKTTCLQECLITTFQDAYFVS
DSFEEAKEKMRDFAKSITRPFSVYFNPYTQSIEILKDTRSIENVVQDLRSDLNTVCDALNKMNQYLGI
>rattus_TpH2 NP_776211.1
MQPAMMMFSSKYWARRGLSLDSAVPEEHQILGGLTQNKATASKSEDKRSGKDTSESSKTAVVFSLKNEVG
GLVRALRLFQEKHVNMLHIESRRSRRRSSEVEIFVDCECGKTEFNELIQLLKFQTTIVTLNPPDNIWTEE
EELEDVPWFPRKISELDRCSHRVLMYGTELDADHPGFKDNVYRQRRKYFVDVAMGYKYGQPIPRVEYTEE
ETKTWGVVFRELSKLYPTHACREYLKNFPLLTKYCGYREDNVPQLEDVSMFLKERSGFTVRPVAGYLSPR
DFLAGLAYRVFHCTQYVRHGSDPLYTPEPDTCHELLGHVPLLADPKFAQFSQEIGLASLGASDEDVQKLA
TCYFFTIEFGLCKQEGQLRAYGAGLLSSIGELKHALSDKACVKAFDPKTTCLQECLITTFQDAYFVSESF

EEAKEKMRDFAKSITRPFSVYFNPYTQSIEILKDTRSIENVVQDLRSDLNTVCDALNKMNQYLGI
>human_TpH1 NP_004170.1
MIEDNKENKDHSLERGRASLIFSLKNEVGGLIKALKIFQEKHVNLLHIESRKSKRRNSEFEIFVDCDINR
EQLNDIFHLLKSHTNVLSVNLPDNFTLKEDGMETVPWFPKKISDLDHCANRVLMYGSELDADHPGFKDNV
YRKRRKYFADLAMNYKHGDPIPKVEFTEEEIKTWGTVFQELNKLYPTHACREYLKNLPLLSKYCGYREDN
IPQLEDVSNFLKERTGFSIRPVAGYLSPRDFLSGLAFRVFHCTQYVRHSSDPFYTPEPDTCHELLGHVPL
LAEPSFAQFSQEIGLASLGASEEAVQKLATCYFFTVEFGLCKQDGQLRVFGAGLLSSISELKHALSGHAK
VKPFDPKITCKQECLITTFQDVYFVSESFEDAKEKMREFTKTIKRPFGVKYNPYTRSIQILKDTKSITSA
MNELQHDLDVVSDALAKVSRKPSI
>mouse_TpH1 NP_001129556.1
MIEDNKENKENKDHSSERGRVTLIFSLENEVGGLIKVLKIFQENHVSLLHIESRKSKQRNSEFEIFVDCD
ISREQLNDIFPLLKSHATVLSVDSPDQLTAKEDVMETVPWFPKKISDLDFCANRVLLYGSELDADHPGFK
DNVYRRRRKYFAELAMNYKHGDPIPKIEFTEEEIKTWGTIFRELNKLYPTHACREYLRNLPLLSKYCGYR
EDNIPQLEDVSNFLKERTGFSIRPVAGYLSPRDFLSGLAFRVFHCTQYVRHSSDPLYTPEPDTCHELLGH
VPLLAEPSFAQFSQEIGLASLGASEETVQKLATCYFFTVEFGLCKQDGQLRVFGAGLLSSISELKHALSG
HAKVKPFDPKIACKQECLITSFQDVYFVSESFEDAKEKMREFAKTVKRPFGLKYNPYTQSVQVLRDTKSI
TSAMNELRYDLDVISDALARVTRWPSV
>rattus_TpH1 NP_001094104.1
MIEDNKENKDHSSERGRVTLIFSLKNEVGGLIKALKIFQENHVNLLHIESRKSKRRNSEFEIFVDCDINR
EQLNDIFPLLKSHTTVLSVDSPDQLPEKEDVMETVPWFPKKISDLDFCANRVLLYGSELDADHPGFKDNV
YRRRRKYFAELAMNYKHGDPIPKIEFTEEEIKTWGTIFRELNKLYPTHACREYLRNLPLLSKYCGYREDN
VPQLEDVSNFLKERTGFSIRPVAGYLSPRDFLSGLAFRVFHCTQYVRHSSDPLYTPEPDTCHELLGHVPL
LAEPSFAQFSQEIGLASLGASEETVQKLATCYFFTVEFGLCKQDGQLRVFGAGLLSSISELRHALSGHAK
VKPFDPKVACKQECLITSFQDVYFVSESFEDAKEKMREFAKTVKRPFGVKYNPYTQSIQVLRDSKSITSA
MNELRHDLDVVNDALARVSRWPSV
>zebrafish_Tph1a NP_840091.1
MYSSKSDGPRRGRSFDSMNLGMTLEEKQLNNEMNKSAFTKIEENKDNKTESSETGRAAVVFSLKNEVGGL
VKALKLFQENHVNLVHIESRKSKRRNSEFEIFVDCDSNREQLHEIIQLLRKHVNVVEMDAPDNRLAEESE
MENVPWFPKKISDLDKCANRVLMYGSDLDADHPGFKDNVYRKRRKYFADLAMSYKHGDPIPRIEFTEEEV
KTWGVVFRELNKLYPSHACREYLKNLPLLIKHCDSREDNIPQLEDVSRFLKERTGFTIRPVAGYLSPRDF
LAGLAFRVFHCTQYVRHSSDPLYTPEPDTCHELLGHVPLLAEPSFAQFSQEIGLASLGASDDSIQKLATC
YFFTVEFGLCKQEGKLRAYGAGLLSSISELKHALSGNARILPFDPNVTCKQECIITTFQDVYFMSDSFEE
AKVKMREFAKTIKRPFSVRYNPYTQSVDVLKDTTLNNVVEELNMTGHLGDA
>zebrafish_TpH1b NP_001001843.2
MLSNKLDGPRRGRSFDSINKNYEEKLLSNELRKTTFHKTDENKDKKLSSKREHAAIVFSLKNEVGGLVKA
LKLFQDNQVNLLHIESRKSRRRNSELEVLVDCDSDRETLKEIVQLLRKQTSIIAMNSPDKFWTPASDLTE
VPWFPKKISDLDKSACRVLMYGSELDADHPGFKDNVYRKRRKYFADLAMSYKHGDPIPHVEFTEEEVKTW
GVVFRELNKLYPTHACREYLQNLPLLSQFCGYREDNIPQLEDVSNFLRERTGFTIRPVAGYLSPRDFLAG
LAFRVFHCTQYVRHSSDPLYTPEPDTCHELLGHVPLLAEPSFAQFSQELGLASLGASDDAVQKLATCYFF
TVEFGLCKQEGSLRAYGAGLLSSISELKHSLSDSAKILPFEPKVTCKQECLITTFQDVYFVSESFEEAKC
RMREFAKTIQRPFSLRYNPYTQSVCVLKDMPSINDVVEELRHELDIVGDALCRLSTHLGV
>zebrafish_Tph2 NP_999960.1
MTNSTLHPPWTSFNRIDERPDKEEQKSTHDLGKLAVIFSLKNEVGFLVKALRLFQEKHVNLAHIESRRSK
RLTNEIEIYAECNCTKKEFNELVQHLKDHVNIVSYNTPQHVWSAETDCLDCVCVLGGLPDGEGIPWFPQK
ISELDQCSHRVLMYGSELDADHPGFKDKVYRQRRKYFVEVAMNYKFGQPIPRIEYTAEEVKTWGVVYREL
TKLYPTHACREYLKNLPLLTKHCGYREDNIPQLEDVSLFLRERSGFTVRPVAGYLSPRDFLAGLAYRVFN
CTQYIRHSTDPLYTPEPDTCHELLGHVPLLADPKFAQFSQEIGLASLGASDEDVQKLATCYFFTIEFGLC
EQDGQLRVYGAGLLSSIGELRHALSDKATVKVFDPKTTCYQECLITTFQDVYFVSESFEEAKEKMGEFAK
SIKRPFSVYYDPYTQSIDLLKDTRSIENCGSRSTQRLT

Serotonin Transporter (SERT) proteins

>human_SERT EAW51223.1
METTPLNSQKQLSACEDGEDCQENGVLQKVVPTPGDKVESGQISNGYSAVPSPGAGDDTRHSIPATTTTLVAELHQGERE
TWGKKVDFLLSVIGYAVDLGNVWRFPYICYQNGGGAFLLPYTIMAIFGGIPLFYMELALGQYHRNGCISIWRKICPIFKG
IGYAICIIAFYIASYYNTIMAWALYYLISSFTDQLPWTSCKNSWNTGNCTNYFSEDNITWTLHSTSPAEEFYTRHVLQIH
RSKGLQDLGGISWQLALCIMLIFTVIYFSIWKGVKTSGKVVWVTATFPYIILSVLLVRGATLPGAWRGVLFYLKPNWQKL
LETGVWIDAAAQIFFSLGPGFGVLLAFASYNKFNNNCYQDALVTSVVNCMTSFVSGFVIFTVLGYMAEMRNEDVSEVAKD
AGPSLLFITYAEAIANMPASTFFAIIFFLMLITLGLDSTFAGLEGVITAVLDEFPHVWAKRRERFVLAVVITCFFGSLVT

LTFGGAYVVKLLEEYATGPAVLTVALIEAVAVSWFYGITQFCRDVKEMLGFSPGWFWRICWVAISPLFLLFIICSFLMSP
PQLRLFQYNYPYWSIILGYCIGTSSFICIPTYIAYRLIITPGTFKERIIKSITPETPTEIPCGDIRLNAV
>mouse_SERT AAB67172.1
METTPLNSQKVLSECKDKEDCQENGVLQKGVPTPADKAEPGQISNGYSAVPSTSAGDEAPHSTPAATTTLVAEIHQGERE
TWGKKMDFLLSVIGYAVDLGNIWRFPYICYQNGGGAFLLPYTIMAIFGGIPLFYMELALGQYHRNGCISIWRKICPIFKG
IGYAICIIAFYIASYYNTIIAWALYYLISSFTDQLPWTSCKNSWNTGNCTNYFAQDNITWTLHSTSPAEEFYLRHVLQIH
QSKGLQDLGTISWQLALCIMLIFTIIYFSIWKGVKTSGKVVWVTATFPYIVLSVLLVRGATLPGAWRGVVFYLKPNWQKL
LETGVWVDAAAQIFFSLGPGFGVLLAFASYNKFNNNCYQDALVTSVVNCMTSFVSGFVIFTVLGYMAEMRNEDVSEVAKD
AGPSLLFITYAEAIANMPASTFFAIIFFLMLITLGLDSTFAGLEGVITAVLDEFPHIWAKRREWFVLIVVITCILGSLLT
LTSGGAYVVTLLEEYATGPAVLTVALIEAVVVSWFYGITQFCSDVKEMLGFSPGWFWRICWVAISPLFLLFIICSFLMSP
PQLRLFQYNYPHWSIILGYCIGTSVICIPIYIIYRLISTPGTLKERIIKSITPETPTEIPCGDIRMNAV
>sheep_SERT NP_001009446.1
METTPLNSQKELSAYKDGEDCQENGVLQKGVPAPGDKAESGQISNGYSAVPNPGAGDDTQHSIPAATTALVAEVHPAERE
TWGKKVDFLLSVIGYAVDLGNVWRFPYICYQNGGGAFLLPYTIMAIFGGIPLFYMELPLGQYHRNGCISIWTKICPIFKG
IGCAICLIAFYIASYYNTIMAWALYYLISSFTEQLPWTSCENSWNTGNCTNYFSEDNITWMLHSTSPAEEFYTRHVLQIH
RSKGLQDLGGLSWQLVLCIMFIFTIIYFSIWKGVKTSGKVVWVTAIFPYIILLILLVRGATLPGAWRGVLFYLKPNWQKL
LETGVWVDAAAQIFFSLGPGFGVLLAFASYNKFHNNCYQDALVTSAVNCMTSFVSGFVIFTVLGYMAEMRKEDVSEVAKD
AGPSLLFITYAEAIANMPASTFFAIVFFLMLITLGLDFTFAGLEGVITAVLDEFPHVWAKRREWFVLGVVITCFFGSLVT
LTFGGAYVVKLLEEFATGPAVLTVALIEAVAVFWFYGINQFCIDVKEMLGFSPGWFWKICWVAISPLFLLFIICSFLMSP
PQLRLFRYDYPRWSIILGYCIGTSSFICIPTYITYRLIVTPGTLKERIIKGITPKTPTEIPCWGHPLEMLC
>chicken_SERT NP_998737.1
MENKATSNETQPLTSKKGISDCNEGEDCKENGLLIRNPKSALRLVDDGNKVHPGQGDKEEAAQISNGYSGVQSSGPCSGM
GEAEDAQCTAPAATTTTTTTSTTCGAEGQQQLMELGDRETWSKKIDFLLSVIGYAVDLGNVWRFPYICYQNGGGAFLIP
YTIMAIFGGIPLFYMELALGQYHRNGCISIWRKICPIFKGIGFAICIIDLYVASYYNTIMAWVFYYLVSSFTTELPWTSC
NNAWNTGNCTTYFSKDNISWALHSISPAEEFYTRQVLQVHRSNGLDDLGGISWQLTLCLLLIFIIVYFSIWKGVKTSGKV
VWVTATFPYVILFILLVRGATLPGAWRGVLYYLKPEWQKLLATEVWVDAAAQIFFSLGPGFGVLLAYASYNKFHNNCYQD
ALVTSTVNCLTSFVSGFVIFTVLGYMAEMRNEDVSEVAKDMGPSLLFITYAEAIANMPASTFFAIIFFLMLLTLGLDSTF
AGLEGVITGVLDEFPHVWSKRREFFVLGLIIICFLGSLATLTFGGAYVVKLFEEYATGPAVLTVVFLEAVAVAWFYGITQ
FCNDVKEMLGFAPGWYWRVCWVAISPIFLLFVTCSFLSNPPELRLFDYNYPYWTTVVGYCIGTSSIICIPIYMAYRLIIT
PGTLKERILKSITPETATEIPFGDIRMNAV
>zebrafish_SERT NP_001035061.1
MDMKESMMMNQEYGGEQQKVPESQENGRLVVDSVPEKDQKSGSGPGQVSNGYRSTSPQSPKEGAGTGTDRVNTPGTFRTL
VVQQTSLDPPRETWSKKMDFLLSVIGYAVDLGNVWRFPYICYQNGGGAFLLPYLLMAVFGGVPLFYMELALGQFHRSGCI
SIWKHVCPIFKGIGFAICIIALYIAFYYNTIMAWALYYLLSSFRATLPWTTCNNRWNTPNCTHYLSTDLNVSWTNNSISP
AEEFYVRQVLQVHLSPGLHQLGWVSWQLALCLLFIFTVVYFSIWKGVKTSGKVVWVTATFPYLVLLILLIRGATLPGAWR
GVVFYLKPDWKKLLTTTVWLDAAAQIFFSLGPGFGVLLAFASYNPFHNNCYKDALITSSVNCLTSFLSGFVIFTVLGYMA
EMRQQGVETVAKDAGPSLLFIIYAEAIANMPAATFFAIIFFLMIIMLGLDSTFAGLEGVITAMLDEFPHLLARRREWFVL
GLVCVCYLGALSTLTYGGAFVVKLFEEYATGPAVITVVFLEVIAVSWFYGTTRFCNDVQLMLGFAPGLFWRVCWIAICPC
FLLFIIVSFLAFPPEVKLFDYLYPFWTTVLGYCIGVSSFICVPSYMVYHLVTTKGTFQQRLLKGITPEAPGSSGPQRDTI
VITNAV
>amphioxus_SERT
MEYNNPVFDRTGTASWHGQETDMAKVSATSSPPSHRDDRAAAPPSSQLHPAAADGDLGKVSLAEDGPDAPADGEERETWS
KKVDFLLSVIGFAVDLGNVWRFPYICHRNGGGAFLVPYFIMVIFGGIPLCYMELSLGQYHRLGPLKIWTKICPLFKGMGY
AMILVGMYTSFYYNTIIAWAVFYFFSSFTSELPWRSCNNTWNTDNCTEFAVDTWRNNSVSAATEFLVRNVYEYQNSNGLS
ELGYIRWQIALCLALVFVIVYFSLWKGIHMSGKVVWVTATLPYVLLLILMVRGVTLPGADRGILYFLTPKWETLASPSVW
WDAAAQVFFSLGPGFGVLITLSSYNRFHNNTCRDAVATSLMNCLASLLSGFVIFSVLGYISVTQNREIENIAVGGPGLLF
EVYAEAIASMEGATGWSLIFFFMLINLGVDSTFGGLETVITSLSDEYPRVLRRHRELFVLGLVCVCYTGALVTTTNGGVL
VVHLMDTFAANTSITIVVLLEVTIVCWIYGTDRLSCEMQEMMGTKPALPWRICWTFICPIILAITIVLGLVYYESPVYGD
YVYPGWAVGVGWGITGSSLVCIPLYAMYKFIMTPGTLQQRARHLLFMEDSEDAPMTEIAENGRASPMEFKGLVAQALPNS
NV
>sea squirt_SERT XP_002125543.1
MNDFGGADPPVEKIGQENWDTFDCSPDPHTPRKASLLSAPGSLQTPQRSNSFPVVHRFTVHNGDSNTLLNGGEAPKRRVS
GYSTTSTEKAKDRKQSNQSTNGYGSPKYRKESTMTNDSLPTSSSTYGLVSSSENKDKRLIPKQGSKSLEREQWGRKLEFL
LSVIGFAVDLGNVWRFPYICFRNGGGAFLIPYFLMIVFGGIPLFYLELILGQYHRTGCISIWGKICPIFKGVGYAICLMA
LYVSSYYNTVIGWAVYYLYSSFAYELPWATCNHSWTDSRCLDKINAAAHNQTWTNDTQSPAQQFFDRHVLEVYKSTGLGD
LGPPRWQIVLCLFVVYFILYFCLWKGVRSSGKVVWVTATFPYIVLFILLIRGAMLPGASIGISYYLSPQWHLLAKPTVWL
EAATQVFFSLGPGFGTLIALSSYNRFDNNCYRDAFITSVVNCLTSFMAGFVVFSVLGYMAHLLNKTDIEEVTTPGVGLLF
VVYGQALTTFSGSVFFSIIFFLMIITLGLDSSFGGLEAVITGFSDEYPETIGKHREKFVLGLLSVSFLLALATTTQGGVY
LMTLLEVYATGPAIMTVVLLESISVTWFYGINRLCEDIKAMLGFGPGIFWRVCWTVISPLFVSFMIVMSFIFTSNLQYGD
YIFPNWSTWIGWAITLSSITIVPVYALYKFIFEPGSPQQRCFSLLRPEVGGQAQPRESLAMTSVPA
> sea slug_SERT NP_001191502.1

MTPGPELPLMDGTPTKLIDKPASKPEPERESWGKKIDFLLSVIGFAVDLGNVWRFPYVCYKNGGGAFLIPYLIMLIFGGL
PLFYMELALGQFQRCGCFTVWKRLCPMLKGIGMAICIIATLVSWYYNTIVAWAVYFLFSSFTNSPPWLSCNNTWNTPNCT
TFSDRILPYTEKCETEVLSYNHTMLNTTAMMNDTEAGVSIHLVNATQYKRAVCRLVKEVDTSGNFYATAASTEFFERNVL
ELQHADGISSVGGVKTTLALCLFGVFFIVYFALWKGIKSSGKAVWITATLPYVVLLILLCKGCTLPGAGDGIVYYLSPQW
EKLLNLEIWIAAAAQIFFSLGPGFGVLLALSSYNKFHNNCYRDALITSATNCLTSFLAGFVVFTVLGYMAHVQHRTVETV
ARQDVGLIFVVYPEAVATLEGTSFWAVIFFFMLIMLGLDTTFGGLEAIITGILDEWTFLRRHRELFVAGLMLWCFLGGLV
TTTYGGIYVIQLMDTYGAPISILLIVFLEAVAVSWIYGVDRFSHDIETMLGTAPGPFWRVSWTYISPLFLLVLFILSLMT
SPPPQYGDYVYPYWSLAVGWLIVCITLVSIPIFIVISFFNSKGTFKERIYQMITPTEVPSHVPKRDAPVYL
>fruit fly_SERT NP_523846.2
MDRSGSSDFAGAAATTGRSNPAPWSDDKESPNNEDDSNEDDGDHTTPAKVTDPLAPKLANNERILVVSVTERTRETWGQK
AEFLLAVIGFAVDLGNVWRFPYICYQNGGGAFLVPYCLFLIFGGLPLFYMELALGQFHRCGCLSIWKRICPALKGVGYAI
CLIDIYMGMYYNTIIGWAVYYLFASFTSKLPWTSCDNPWNTENCMQVTSENFTELATSPAKEFFERKVLESYKGNGLDFM
GPVKPTLALCVFGVFVLVYFSLWKGVRSAGKVVWVTALAPYVVLIILLVRGVSLPGADEGIKYYLTPEWHKLKNSKVWID
AASQIFFSLGPGFGTLLALSSYNKFNNNCYRDALITSSINCLTSFLAGFVIFSVLGYMAYVQKTSIDKVGLEGPGLVFIV
YPEAIATMSGSVFWSIIFFLMLITLGLDSTFGGLEAMITALCDEYPRVIGRRRELFVLLLLAFIFLCALPTMTYGGVVLV
NFLNVYGPGLAILFVVFVEAAGVFWFYGVDRFSSDVEQMLGSKPGLFWRICWTYISPVFLLTIFIFSIMGYKEMLGEEYY
YPDWSYQVGWAVTCSSVLCIPMYIIYKFFFASKGGCRQRLQESFQPEDNCGSVVPGQQGTSV
> nematode worm_SERT AAK84832.1
MLRWHSVRRKQHQQLQAELSSGAASMLSAPESRRVSRSMSVKAPTASEYMPLSVADKPLTLTVSTSHSIDPNEPIAALGG
LTPTKEGRVAALRRRSSMVRDKWATKMEFLLAVVGYAVDLGNIWRFPSVCYKHGGGAFLIPYFIMLMIGGLPMFYMELVL
GQFHRSGCVSIWRKVCPLFRGIGYGICCICTFIAIFYNAIIAQAVYFAIVSLSKIWDSEVPWASCGNPWNTPRCSDDLNV
TISRNGTPLTTPSEEYYLYKVLEVQKSTGFDDLGGVKTSMAVCLLAVFIMVYFALWKGPQSSGKIVWVTATAPYIILSIL
LIRGLLLPGAKNGLYYYVTPDFEKLKDPAVWSAAATQIFFSLGPGFGVLLALSSYNDFNNNCYRDAVTISIINCATSFFS
GCVVFSTLGYMSLLTNKPINEVVGEHDASLIFIVYPQALATMDYSCFWSFIFFVMLITLGIDSTFAGIEAFITGFCDESR
FLSKNRKWFVLVICIIYYFLSFPAISYGGQFVIPFLDEYGVSLSVLFIVTCEMIAVCWFYGVDQFSKDIRAMLGFYPGIY
WRVCWTCSPVFISVIFIMTVYNSSFKPIQMASYTFPWWSVILGWFLRLLSVLAIPVFAIIYLLSGTGTLYERFRWAITPQ
QRRNSATSLAADPTQIIDSSLLDPIHTLTPV

Glutamic Acid Decarboxylase (GAD) proteins

>human_GAD65 AAA62367.1
MASPGSGFWSFGSEDGSGDSENPGTARAWCQVAQKFTGGIGNKLCALLYGDAEKPAESGGSQPPRAAARK
AACACDQKPCSCSKVDVNYAFLHATDLLPACDGERPTLAFLQDVMNILLQYVVKSFDRSTKVIDFHYPNE
LLQEYNWELADQPQNLEEILMHCQTTLKYAIKTGHPRYFNQLSTGLDMVGLAADWLTSTANTNMFTYEIA
PVFVLLEYVTLKKMREIIGWPGGSGDGIFSPGGAISNMYAMMIARFKMFPEVKEKGMAALPRLIAFTSEH
SHFSLKKGAAALGIGTDSVILIKCDERGKMIPSDLERRILEAKQKGFVPFLVSATAGTTVYGAFDPLLAV
ADICKKYKIWMHVDAAWGGGLLMSRKHKWKLSGVERANSVTWNPHKMMGVPLQCSALLVREEGLMQNCNQ
MHASYLFQQDKHYDLSYDTGDKALQCGRHVDVFKLWLMWRAKGTTGFEAHVDKCLELAEYLYNIIKNREG
YEMVFDGKPQHTNVCFWYIPPSLRTLEDNEERMSRLSKVAPVIKARMMEYGTTMVSYQPLGDKVNFFRMV
ISNPAATHQDIDFLIEEIERLGQDL
>human_GAD67 AAA62368.1
MASSTPSSSATSSNAGADPNTTNLRPTTYDTWCGVAHGCTRKLGLKICGFLQRTNSLEEKSRLVSAFRER
QSSKNLLSCENSDRDARFRRTETDFSNLFARDLLPAKNGEEQTVQFLLEVVDILLNYVRKTFDRSTKVLD
FHHPHQLLEGMEGFNLELSDHPESLEQILVDCRDTLKYGVRTGHPRFFNQLSTGLDIIGLAGEWLTSTAN
TNMFTYEIAPVFVLMEQITLKKMREIVGWSSKDGDGIFSPGGAISNMYSIMAARYKYFPEVKTKGMAAVP
KLVLFTSEQSHYSIKKAGAALGFGTDNVILIKCNERGKIIPADFEAKILEAKQKGYVPFYVNATAGTTVY
GAFDPIQEIADICEKYNLWLHVDAAWGGGLLMSRKHRHKLNGIERANSVTWNPHKMMGVLLQCSAILVKE
KGILQGCNQMCAGYLFQPDKQYDVSYDTGDKAIQCGRHVDIFKFWLMWKAKGTVGFENQINKCLELAEYL
YAKIKNREEFEMVFNGEPEHTNVCFWYIPQSLRGVPDSPQRREKLHKVAPKIKALMMESGTTMVGYQPQG
DKANFFRMVISNPAATQSDIDFLIEEIERLGQDL
>pig_GAD65 BAA06635.1
MASPGSGFWSFGSEDGSGDPENSGTARAWCQVAQKFTGGIGNKLCALLYGDAEKPAESGGSQPPRTTSRK
ATCACNQKPCNCPKAEVNYAFLHATDLLPACDGERPTLAFLQDVMDILLQYVVKSFDRSTKVIDFHYPNE
LLQEYNWELADQPQNLEEILMHCQTTLKYAIKTGHPRYFNQLSTGLDMVGLAADWLTSTANTNMFTYEIA
PVFVLLEYVTLKKMREIIGWPGGSGDGIFSPGGAISNMYAMLIARFKMFPEVKEKGMAAVPRLIAFTSEH
SHFSLKKGAAALGIGTDSVILIKCDERGKMIPSDLERRILEAKQKGFVPFLVSATAGTTVYGAFDPLLAV
ADICKKYKIWMHVDAAWGGGLLMSRKHKWKLSGVERANSVTWNPHKMMGVPLQCSALLVREEGLMQSCNQ
MHASYLFQQDKHYDLSYDTGDKALQCGRHVDVFKLWLMWRAKGTTGFEAHIDKCLELAEYLYNIIKNREG
YEMVFDGKPQHTNVCFWYVPPSLRVLDNNEERMSRLSKVAPVIKARMMESGTTMVSYQPLGDKVNFFRMV
ISNPAATHQDIDFLIEEIERLGQDL

>pig_GAD67 BAA06636.1
MASSTPSSSATSSNAGPDPNTTNLRPTTYDTWCGVAHGCTRKLGLKICGFLQRTNSLEEKSRLVSAFKER
QSSKNLLSCENSDRDGRFRRTETDFSNLFARDLLPAKNGEEQTVQFLLEVVDILLNYVRKTFDRSTKVLD
FHHPHQLLEGMEGFNLELSDHPESLEQILVDCRDTLKYGVRTGHPRFFNQLSTGLDIIGLAGEWLTSTAN
TNMFTYEIAPVFVLMEQITLKKMREIVGWSNKDGDGIFSPGGAISNMYSIMAARYKYFPEVKTKGMAAVP
KLVLFTSEHSHYSIKKAGAALGFGTDNVILIKCNERGKIIPADLEAKILEAKQKGYIPLYVNATAGTTVY
GAFDPIQEIADICEKYNLWLHVDAAWGGGLLMSRKHRHKLSGIERADSVTWNPHKMMGVLLQCSAILVKE
KGILQGCNQMCAGYLFQPDKQYDVSYDTGDKAIQCGRHVDIFKFWLMWKAKGTVGFENQINKCLELAEYL
YAKIKNREEFEMVFDGEPEHTNVCFWYIPQSLRGVPDSPERREKLHRVAPKIKALMMESGTTMVGYQPQG
DKANFFRMVISNPAATQSDIDFLIEEIERLGQDL
>mouse_GAD65 AAA93049.1
MASPGSGFWSFGSEDGSADPENPGTARAWCQVAQKFTGGIGNKLCALLYGDSGKPAEGGGSVTSRAATGK
VACTCDQKPCNCPKGDVNYAFLHATDLLPACDGERPTLAFLQDVMNILLQYVVKSFDRSTKVIDFHYPNE
LLQEYNWELADQPQNLEEILTHCQTTLKYAIKTGHPRYFNQLSTGLDMVGLAADWLTSTANTNMFTYEIA
PVFVLLEYVTLKKMREIIGWPGGSGDGIFSPGGAISNMYAMLIARYKMFPEVKEKGMAAVPRLIAFTSEH
SHFSLKKGAAALGIGTDSVILIKCDERGKMIPSDLERRILEVKQKGFVPFLVSATAGTTVYGAFDPLLAV
ADICKKYKIWMHVDAAWGGGLLMSRKHKWKLSGVERANSVTWNPHKMMGVPLQCSALLVREEGLMQSCNQ
MHASYLFQQDKHYDLSYDTGDKALQCGRHVDVFKLWLMWRAKGTTGFEAHIDKCLELAEYLYTIIKNREG
YEMVFDGKPQHTNVCFWFVPPSLRTLEDNEERMSRLSKVAPVIKARMMEYGTTMVSYQPLGDKVNFFRMV
ISNPAATHQDIDFLIEEIERLGQDL
>mouse_GAD67 CAA90277.1
MASSTPSPATSSNAGADPNTTNLRPTTYDTWCGVAHGCTRKLGLKICGFLQRTNSLEEKSRLVSAFRERQ
SSKNLLSCENSDQGARFRRTETDFSNLFAQDLLPAKNGEEQTAQFLLEVVDILLNYVRKTFDHSTKVLDF
HHPHQLLEGMEGFNLELSDHPESLEQILVDCRDTLKYGVRTGHPRFFNQLSTGLDIIGLAGEWLTSTANT
NMFTYEIAPVFVLMEQITLKKMREIVGWSNKDGDGIFSPGGAISNMYTIMAARYKYFPEVKTKGMAAVPK
LVLFTSEHSHYSIKKAGAALGFGTDNVILIKCNERGKIIPADLEAKILDAKQKGYVPLYVNATAGTTVYG
AFDPIQEIASICEKYNLWLHVDAAWGGGLLMSRKHRHKLSGIERANSVTWNPHKMMGVLLQCSAILVKEK
GILQGCNQMCAGYLFQPDKQYDVSYDTGDKAIQCGRHVDINKFWLMWKAKGTVGFENQINKCLELADYLY
AKIKNREEFEMVFDGEPEHTNVCFWYIPQSLRGVPDSPERREKLHRVAPKIKALMMESGTTMVAYQPQGD
KANFFRMVISNPAASQSDIDFLTEEIERLGQDL
>rattus_GAD65 AAA63488.1
MASPGSGFWSFGSEDGSGDPENPGTARAWCQVAQKFTGGIGNKLCALLYGDSEKPAESGGSVTSRAATRK
VACTCDQKPCSCPKGDVNYALLHATDLLPACEGERPTLAFLQDVMNILLQYVVKSFDRSTKVIDFHYPNE
LLQEYNWELADQPQNLEEILTHCQTTLKYAIKTGHPRYFNQLSTGLDMVGLAADWLTSTANTNMFTYEIA
PVFVLLEYVTLKKMREIIGWPGGSGDGIFSPGGAISNMYAMLIARYKMFPEVKEKGMAAVPRLIAFTSEH
SHFSLKKGAAALGIGTDSVILIKCDERGKMIPSDLERRILEVKQKGFVPFLVSATAGTTVYGAFDPLLAV
ADICKKYKIWMHVDAAWGGGLLMSRKHKWKLNGVERANSVTWNPHKMMGVPLQCSALLVREEGLMQSCNQ
MHASYLFQQDKHYDLSYDTGDKALQCGRHVDVFKLWLMWRAKGTTGFEAHIDKCLELAEYLYNIIKNREG
YEMVFDGKPQHTNVCFWFVPPSLRVLEDNEERMSRLSKVAPVIKARMMEYGTTMVSYQPLGDKVNFFRMV
ISNPAATHQDIDFLIEEIERLGQDL
>rattus_GAD67 CAA40800.1
MASSTPSPATSSNAGADPNTTNLRPTTYDTWCGVAHGCTRKLGLKICGFLQRTNSLEEKSRLVSAFRERQ
ASKNLLSCENSDPGARFRRTETDFSNLFAQDLVPAKNGEEQTVQFLLEVVDILLNYVRKTFDRSTKVLDF
HHPHQLLEGMEGFNLELSDHPESLEQILVDCRDTLKYGVRTGHPRFFNQLSTGLDIIGLAGEWLTSTANT
NMFTYEIAPVFVLMEQITLKKMREIIGWSNKDGDGIFSPGGAISNMYSIMAARYKYFPEVKTKGMAAVPK
LVLSTSADSHYSIKKAGAALGFGTDNVILIKCNERGKIIPADLEAKILDAKQKGFVPLYVNATEATIVYG
ALEPIQEIADICEKYNLWLHVDAAWGGGLRMSRKHRHKLSGIERANSVTWNPHKMMGVLLQCSAILVKEK
GILQGCNQMCAGYLFQPDKQYDVSYDTGDKAIQCGRHVDIFKFWLMWKAKGTVGFENQINKCLELAEYLY
AKIKNREEFEMVFNGEPEHTNVCFWYIPQSLRGVPDSPERREKLHRVAPKIKALMMESGTTMVGYQPQGD
KANFFRMVISNPAATQSDIDFLIEEIERLGQDL
>zebrafish_GAD67 NP_919400.1
MASSAPSSSAGDMDPNTANLRQPATTSDAWYGVAHGCTRKLGMKICGFLQKNNSLDEKSRMVGSFKESAK
NQMSCDNNERFTRDETDFSNLFARDLLPAKNGEEPTIQFLLEVVEILTNYVRKTFDRSTKVLDFHHPHQL
LEGMEGFNLELSDQPESLEQILVDCRDTLKYGVRTGHPRFFNQLSSGLDIIGLAGEWLTSTANTNMFTYE
IAPVFVLMEQLTLKKMREIIGWPNGDGDALFSPGGAISNMYSVMVARYKYFPEVKTKGMSAAPRLVLFTS
EHSHYSIKKAGAVLGFGKENVILLKTDERGRVIPADLEAKVIDAKQKGYVPLFVNATAGTTVYGAFDPIN
DIADICEKYNLWLHVDGAWGGGLLMSRKHRHKLSGIERANSVTWNPHKMMGVPLQCSAILVREKGILQGC
NSMCAGYLFQPDKQYDVTYDTGDKAIQCGRHVDIFKFWLMWKAKGTIGFEQHIDRCLELSEYLYNKIKNR
EGYEMVFEGQPQHTNVCFWYIPPSLRGMPNGDERREKLHRVAPKIKAMMMECGTTMVGYQPQGDKVNFFR
MVVSNHAVTKSDIDFLIDEIERLGQDL
>zebrafish_GAD65 NP_001017708.2

MASHGFWFLGAENAAGNGSQSPNTPRAWCQAAAQKFSGGIGSKLCALLNVGEAEKAAQAPVKAEDESTAE
SCGCNKPCNCSKATACFSDLYSTDLLPALDGDAKTMNFLQEVVDILLAYIVESFDRSTKVIDFHYPNELL
QRNNWELSDEPETLDDILISCRATLKYAIKTAHPRYFNQLSTGLDMVGLAADWLTSTANTNMFTYEVAPV
FVLLEYVTLKKMREIIGWQDGHGDGIFSPGGAISNMYAMLLARYKMFPEVKEKGMSSVPRLVAFTSEHSH
FSIKKGAAALGIGTESVICIKADERGKMIPSDLERRIIEAKQKGYVPFFVSATAGTTVYGAFDPLIAIAD
ICKKHDVWMHVDGAWGGSLLMSRKHRWKLNGVERANSMTWNPHKMMAVPLQCSALLVREEGLMQSCNQMQ
ACYLFQQDKHYDLQYDTGDKALQCGRHVDIFKLWLMWRAKGTIGFEAQIDKCLELSEYLYNKIKDREGYQ
MVFDGKPQHTNVCFWYLPPGVRYLEDKVERMKRLHKVAPVIKARMMEYGTTMVSYQPQGDKVNFFRMVIS
NPAATFEDIDFLIEEIERLGQDL
>nematode worm_GAD NP_499689.1
MSSAAADESDAVLENLIAKEILPQTGNWEGTEEFLNRIVQVLLKYIKDQNDRDQKILEFHHPDKMQMLMD
LSIPEKPESLLKLVKSCEDVLRLGVRTGHPRFFNQISCGLDLVSMAGEWLTATANTNMFTYEIAPVFILM
EKSVMARMWEAVGWDPEKADGIFAPGGAIANLYAMNAARHQLWPRSKHLGMKDIPTLCCFTSEDSHYSIK
SASAVLGIGADYCFNIPTDKNGKMIPEALEAKIIECKKEGLTPFFACCTAGSTVYGAFDPLERVANICER
HKLWFHVDAAWGGGMLLSPEHRYKLAGIERANSVTWNPHKLMGALLQCSACLFRQDGLLFQCNQMSADYL
FQQDKPYDVSFDTGDKAIQCGRHNDVFKLWLMWKSKGMEGYRQQINKLMDLANYFTRRIKETEGFELIIE
NPEFLNICFWYVPSKIRNLEPAEMRARLEKIAPKIKAGMMQRGTTMVGYQPDKQRPNFFRMIISNQAITR
EDLDFLIKEIVDIGESLE
>fruit fly_GAD CAA53791.1
MSLNPNGYKLSERTGKLTAYDLMPTTVTAGPETREFLLKVIDVLLDFVKATNDRNEKVLDFHHPEDMKRL
LDLDVPDRALPLQQLIEDCATTLKYQVKTGHPHFFNQLSNGLDLISMAGEWLTATANTNMFTYEIAPVFI
LMENVVLTKMREIIGWSGGDSILAPGGSISNLYAFLAARHKMFPNYKEHGSVGLPGTLVMLTSDQCHYSI
KSCAAVCGLGTDHCIVVPSDEHGKMITSELERLILERKAKGDIPFFVNATAGTTVLGAFDDINTIADICQ
KYNCWMHIDAAWGGGLLMSRTHRHPRFTGVERADSVTWNPHKLMGALLQCSTIHFKEDGLLISCNQMSAE
YLFMTDKQYDISYDTGDKVIQCGRHNDIFKLWLQWRAKGTEGFEQQQDRLMELVQYQLKRIREQSDRFHL
ILEPECVNVSFWYVPKRLRGVPHDAKKEVELGKICPIIKGRMMQKGTLMVGYQPDDRRPNFFRSIISSAA
VNEADVDFMLDEIHRLGDDL
>sea squirt_GAD NP_001027785.1
MQKQSAWRFRSSSVGLIKVEPNNDDYSMNNGDFENSSSADLLPFGKSDEKTQLFLKEIFEILLKYISKSF
DRKCKILDFHHPHQLLEGIEGFSLNINGEAESLEQILVDCRDTLKYGVKTGHPRFFNQLSSGLDIVSLAA
DWVTSTANTNMFTFEIAPVFILMEDVIIKRMMKIIGWENIDGIFSPGGSINNLYSVMLARHKIMPDVKHS
GLRGFPQLVMFQSKHAHYSNKRPAAILGIGLNNCIDIEVDERGHMKPEDLELKILQSKLDGKVPFYVTAT
AGTTVRGAFDEIVKISEVCKKYKIWLHVDAAWGGAVMMSQKHRHLVAGIEMSDSVTWNPHKMVGVVLQCS
MLLTKHKRLLESCNNMRADYLFQQDKHYDITYDTGDKTIQCGRHVDVFKLWLSWRAKGDKGFCHHVERCI
ELARYLVRKIKKTPGFQLVFQEPEYSNVCFWYYPPSIRNICDEVIKNEKLGKVAPIIKSRMMERGSIMIG
YQPLGSKVNFFRCVISNCAVNYDDIDFMVGQIERLGHDIDM
>amphioxus_GAD
MRTARVPQMTRFRTKSVDLGFLHRDNTISEEDDMPAGKDALKGRSESLEGPDNIIGEESRNSFDFETAYA
RDLLPTRNAPATTTLFLTKVVEILCDYVRKTYDRSEKVVDFHHPQDLKKFLDLEIPDERPETLEKILESC
RETLKYGVKTGHPRFFNQLSSGMDVISLAGEWLTATANTNMFTYEIAPVFIVMEEMILKKMREIIGFPAK
SGDGIFSPGGAISNLYAVNCARYKFVPDVKKKGLREAPKLVMYISEHSHYSLKRAAAIVGIGTDNVYAVK
CDDRGKMIPSDLERKIQIAKSKGETPFFVSASGGSTVYGAFDPLHDLADICQRHKMWLHVDCAWGGGVLL
SKKYRKRLDGIERADSVTWNPHKLMGVILQCSCLLLKESNLLQRCNSMCADYLFQQDKNYDVSYDTGDKT
IQCGRHVDVFKLWLMWRAKATVGFEAQINKCFDLAHYMTDKLKAREGFEMVVDEPELTNICFWYLPPSIR
DLPDGPDKRDRLAQVAPVVKRMMDRGMTMIGYQPLGDNVNFFRMVISNPAATTADIDYMIEEIERLGQDL

GABA/Glycine Transporter (VGAT) proteins

>nematode worm_VGAT NP_499255.1
MASNRFQNLQNWTNKHVFSNSLDYWNQELNEVPSYQNQPQTGESGSNPPPHDRLEPIQESVVSEQPQKDDINKQEEAKDD
GHGEASEPISALQAAWNVTNAIQGMFIVGLPIAVKVGGWWSIGAMVGVAYVCYWTGVLLIECLYENGVKKRKTYREIADF
YKPGFGKWVLAAQLTELLSTCIIYLVLAADLLQSCFPSVDKAGWMMITSASLLTCSFLDDLQIVSRLSFFNAISHLIVNL
IMVLYCLSFVSQWSFSTITFSLNINTLPTIVGMVVFGYTSHIFLPNLEGNMKNPAQFNVMLKWSHIAAAVFKVVFGMLGF
LTFGELTQEEISNSLPNQSFKILVNLILVVKALLSYPLPFYAAVQLLKNNLFLGYPQTPFTSCYSPDKSLREWAVTLRII
LVLFTLFVALSVPYLVELMGLVGNITGTMLSFIWPALFHLYIKEKTLNNFEKRFDQGIIIMGCSVCISGVYFSSMELLRA
INSADS
>fruit fly_VGAT NP_610938.1
MSFIAKLKATPLPPLRNILNVAVQTARQQIPERKDYEQPPGSTAQQHHHSQQAQHKAMEAGMDGGDTTEMSSNPFRNAGS
WTNDGEGGGDGDGEYRNEYQSTSFNEYDGRYQQTDGFRQGSIASEGSSFVCEGEGGGGCKIDEFQAAWNVTNAIQGMFIV
SLPFAVLHGGYWAIVAMVGIAHICCYTGKVLVQCLYEPDPATGQMVRVRDSYVAIAKVCFGPKLGARAVSIAQLIELLMT

CILYVVVCGDLLAGTYPQGSFDSRSWMLFVGIFLLPMGFLKSLKMVSTLSFWCTMSHIVINAVILGYCLLQIGDWGWSKV
RFSIDMENFPISLGVIVFSYTSQIFLPTLEGNMIDRSKFNWMLDWSHIAAAVFKAGFGYICFLTFQNDTQQVITNNLHSQ
GFKGMVNFFLVIKALLSYPLPYYAACELLERNFFRGPPKTKFPTIWNLDGELKVWGLGFRVGVIVSTILMAIFIPHFSIL
MGFIGSFTGTMLSFIWPCYFHIKIKGHLLDQKEIAKDYLIIGLGVLFGVIGIYDSGNALINAFEIGLPF
>sea urchin_VGAT XP_001184388.1
MAATRTTKNLVKEVLKDEDVLAHIKGAIWKALDTKFDELIKRLDIQDGAILDLHNRVKSIESSLTNMQKKQQKCEERNRW
LEKQLNDQEQYSRRNCIRLFGAPEKENENTSQRGMFLVALPYAVMHGGYWTVLSLVLAAIITCYTGLILVDCLYDTNAIT
GERVRVRETYVSIAEEVWGKRFASRVVHTAQFIELIMTCILYLVLCGDLLYNTIRHTPLRESAWTLIACFLVLPCAFLRN
LKAVSRSSFGNAIAHVIINVIILGFCFAQARHWHWKDTSLRIHIHYFPVSLGIVVFSYTSHIFLPSLEGNMVDRRYFKRM
MLWTHGLAGFFKAFFGYVAYLTFGLSTQEVISDNLPTHSFRSIVNLVLVAKALLSFPLPYFAAVELLERAFFQGRPTTVL
PSCYSHDGMLTVWSIPLRLLLICVSVLLAVFIPHFAILMGLIGSVTGTMLSFIWPCWFHLRLKWHELKLWNKVIDILIML
AGAGCGCIGIIYSFEALVKTYVES
>sea squirt_VGAT NP_001027745.1
MAAILDKVRSRIVSLGPTSEEKFSFAKSSDQPHPSGSGCQGDGATNPSSNSVSHPERSTSGVEKPTITAWDAGWNVSNAI
QGMFVLGLPYAVLHGGYLGLILIIVTAVVCCYTGNILIDCLYETSPSGERLRVRSTYVDLAAHCWGKHLGGYLVNAAQLI
ELLMTCVLYVVVSGNLMTNSFPHGPIREAGWSVLACLVLFPCIFLRHLRAVSRFSMGCSVAQIVVLGITIVYCITKINTW
AWSEITISVDMKQFPVSIGVIVFSYTSQIFLPSLEGSMENRGDFRSMLSWSYVASCVTKASFALICFLTWSKDTKDVVTD
NLPPTLRAMINVLLVVKALLSYPLPYYQAIEVMEQTMFTGATGGWGSLFGTKRHAYGEFTDDTEPIVQSTSFNTDAPSSP
SSTTDNSEDGLEDKSTTKNVILTLEDDTNKQSSCPSCYSATGDLQVWALVLRAGLVLGTLLMGVFIPHFALLMGLTGSLT
GTSLAFLFPCAFHLQIKWREMKWREIGLDVFIFISGTVCGITGIYFSIQGLYEVYNPSQISNALGAVNGTSNVTHTALIA
PGFFPEFPLQPDLDLPAFPEEPFISDNSPTSGNKKTNDDTMTYLEESDVIDLVPPPQLPDSAKTSRISEEAQHLRKRR
>amphioxus_VGAT
MAWRWKLTELTRRMVSGPTEENVNFVHFDNLEDGGKYQGTGEVVCLEELGPQKLNGGLQPQEGGAAAGTGAGAGAVAGAG
GGAGTSAATAPPDGTGEGAVVHKPKITAWDAGWNVTTVIQGMFVLSLPYSVVHGGYWSILAIIFVAYVCAYTSKVLVQCL
YEENEKGERIRVRDSYVEIAQAVWGEKTGSRIINVAQFIELTMICILYIVVSGNLLVNSFPHWPIPEQGWSIISTAFLVP
CAFLRHLKGVSRISFYCTIVHLLINACIIGYCFSRAPQWAWDHVTFYINVKMFPVSLGVIVFSYTSQIFLPSLEGNMENR
GNFTTMVNWTHITAGIFKSIFAYICFLTWAETTQEVITDNLPNMAFRALVNVLLTAKALLSYPLPYYQAVELIERDFFQG
HDLTRFPSCYATDGMLKVWALAVRCLLVVGTLLMAVYIPHFALLMGFIGSFTGTLLSFVCPCWFHMKLKWDQISWKIRIW
DCIVIALGTTCGLIGIYYSLEGLIEKFRHDLGYDLND
> African clawed frog_VGAT NP_001079961.1
MATLIRSKLSNVATSVSNKSQAKVSGMFARMGFQAATDEEALGFAHCDDLDTEHRQGLQMDILKTEVPTGDAPPEGDIHY
QRDGTGLPPSASKDEGLCSELSSSEKPQITAWEAGWNVTNAIQGMFVLGLPYAILHGGYLGLFLIIFAAVVCCYTGKILI
ACLYEENEDGETVRVRDSYVDIANACCAPRFPKLGGRVVNVAQIIELVMTCILYVVVSGNLMYNSFPNLPISQKSWSIMA
TAVLLPCAFLKNLKAVSKFSLLCTVAHFVINILVIAYCLSRARDWAWDKVKFYIDVKKFPISIGIIVFSYTSQIFLPSLE
GNMQSPREFHCMMNWTHIAACILKGLFALVAYLTWADETKEVITDNLPSTIRAVVNLFLVSKALLSYPLPFFAAVEVLEK
SLFQEGARAFFPNCYGGDGRLKSWGLTLRCALVVFTLLMAIYVPHFALLMGLTGSLTGAGLCFLLPSLFHLKLMWRQLLW
HQVFFDVSIFVIGSICSVSGFVHSLEGLIEAYAYNIED
>mouse_VGAT NP_033534
MATLLRSKLTNVATSVSNKSQAKVSGMFARMGFQAATDEEAVGFAHCDDLDFEHRQGLQMDILKSEGEPCGDEGAEAPVE
GDIHYQRGGAPLPPSGSKDQAVGAGGEFGGHDKPKITAWEAGWNVTNAIQGMFVLGLPYAILHGGYLGLFLIIFAAVVCC
YTGKILIACLYEENEDGEVVRVRDSYVAIANACCAPRFPTLGGRVVNVAQIIELVMTCILYVVVSGNLMYNSFPGLPVSQ
KSWSIIATAVLLPCAFLKNLKAVSKFSLLCTLAHFVINILVIAYCLSRARDWAWEKVKFYIDVKKFPISIGIIVFSYTSQ
IFLPSLEGNMQQPSEFHCMMNWTHIAACVLKGLFALVAYLTWADETKEVITDNLPGSIRAVVNLFLVAKALLSYPLPFFA
AVEVLEKSLFQEGSRAFFPACYGGDGRLKSWGLTLRCALVVFTLLMAIYVPHFALLMGLTGSLTGAGLCFLLPSLFHLRL
LWRKLLWHQVFFDVAIFVIGGICSVSGFVHSLEGLIEAYRTNAED
>rattus_VGAT NP_113970.1
MATLLRSKLTNVATSVSNKSQAKVSGMFARMGFQAATDEEAVGFAHCDDLDFEHRQGLQMDILKSEGEPCGDEGAEPPVE
GDIHYQRGGAPLPPSGSKDQAVGAGGEFGGHDKPKITAWEAGWNVTNAIQGMFVLGLPYAILHGGYLGLFLIIFAAVVCC
YTGKILIACLYEENEDGEVVRVRDSYVAIANACCAPRFPTLGGRVVNVAQIIELVMTCILYVVVSGNLMYNSFPGLPVSQ
KSWSIIATAVLLPCAFLKNLKAVSKFSLLCTLAHFVINILVIAYCLSRARDWAWEKVKFYIDVKKFPISIGIIVFSYTSQ
IFLPSLEGNMQQPSEFHCMMNWTHIAACVLKGLFALVAYLTWADETKEVITDNLPGSIRAVVNIFLVAKALLSYPLPFFA
AVEVLEKSLFQEGSRAFFPACYGGDGRLKSWGLTLRCALVVFTLLMAIYVPHFALLMGLTGSLTGAGLCFLLPSLFHLRL
LWRKLLWHQVFFDVAIFVIGGICSVSGFVHSLEGLIEAYRTNAED
>human_VGAT NP_542119.1
MATLLRSKLSNVATSVSNKSQAKMSGMFARMGFQAATDEEAVGFAHCDDLDFEHRQGLQMDILKAEGEPCGDEGAEAPVE
GDIHYQRGSGAPLPPSGSKDQVGGGGEFGGHDKPKITAWEAGWNVTNAIQGMFVLGLPYAILHGGYLGLFLIIFAAVVCC
YTGKILIACLYEENEDGEVVRVRDSYVAIANACCAPRFPTLGGRVVNVAQIIELVMTCILYVVVSGNLMYNSFPGLPVSQ
KSWSIIATAVLLPCAFLKNLKAVSKFSLLCTLAHFVINILVIAYCLSRARDWAWEKVKFYIDVKKFPISIGIIVFSYTSQ
IFLPSLEGNMQQPSEFHCMMNWTHIAACVLKGLFALVAYLTWADETKEVITDNLPGSIRAVVNIFLVAKALLSYPLPFFA
AVEVLEKSLFQEGSRAFFPACYSGDGRLKSWGLTLRCALVVFTLLMAIYVPHFALLMGLTGSLTGAGLCFLLPSLFHLRL
LWRKLLWHQVFFDVAIFVIGGICSVSGFVHS
