# Supplementary material for: A neurochemical map of the developing amphioxus nervous system
Source: BMC Neurosci. 2012 Jun 7;13:59. doi: 10.1186/1471-2202-13-59 (PMC3484041; doi:10.1186/1471-2202-13-59)

Phylogenetic trees inferred with Vesicular Glutamate Transporter (VGLUT) proteins

ML

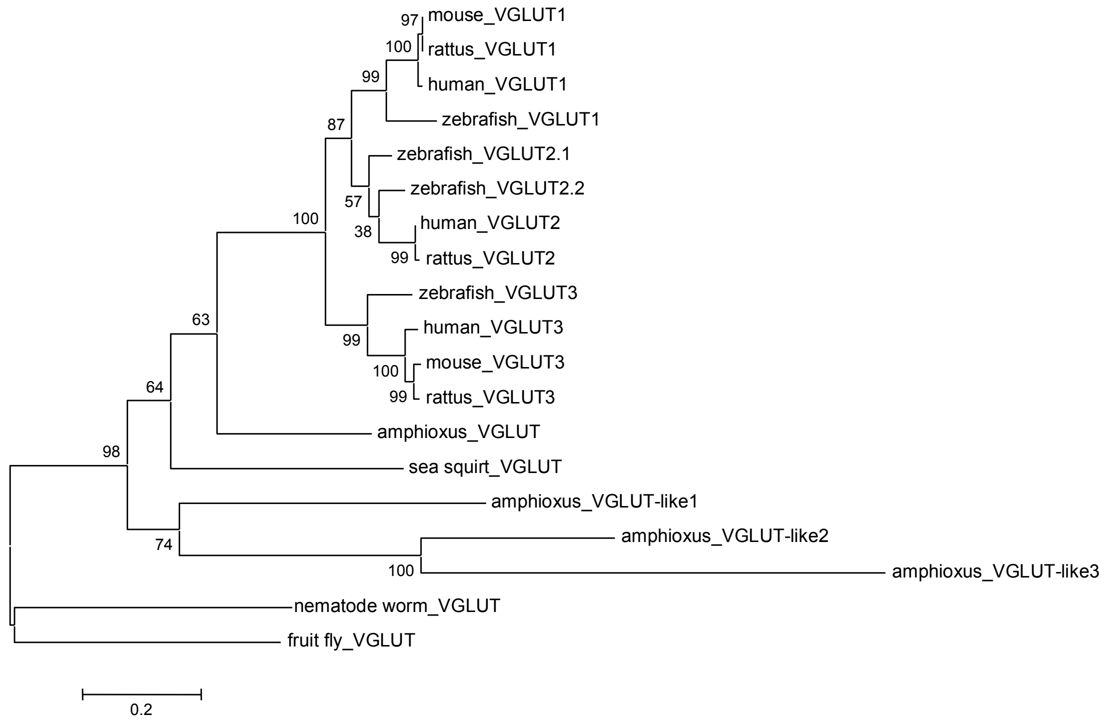

NJ

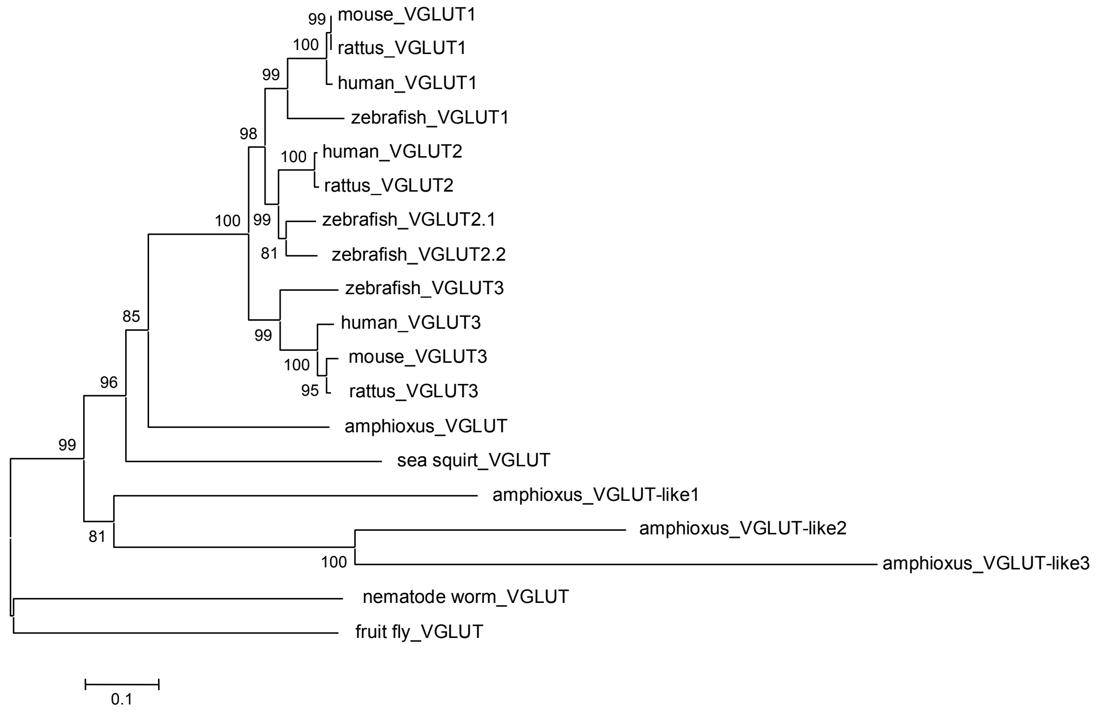

Phylogenetic trees inferred with Tryptophan Hydroxylase (TpH) proteins

ML

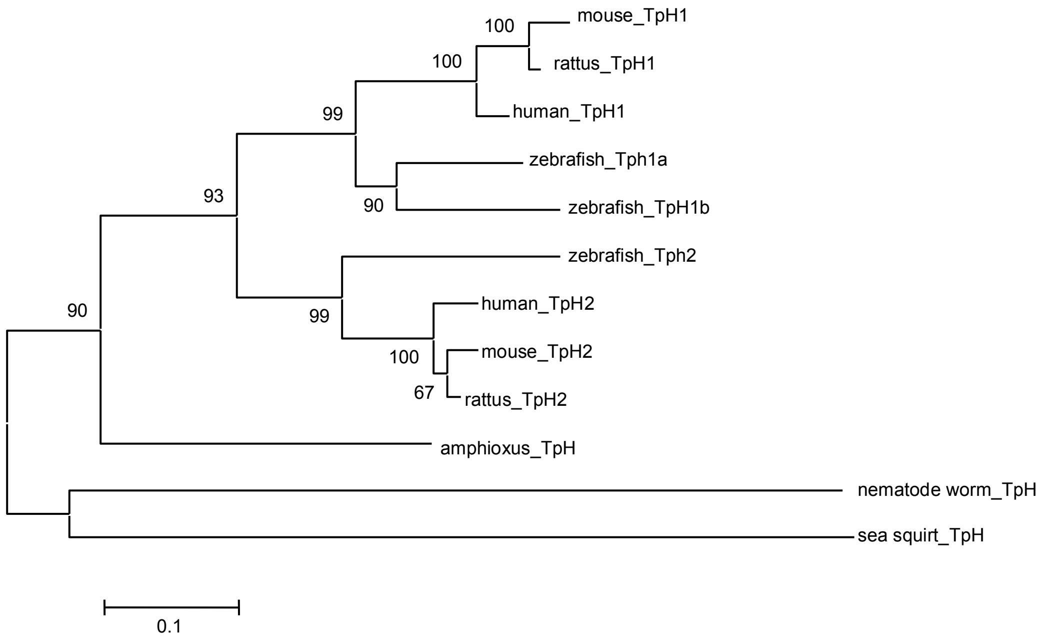

NJ

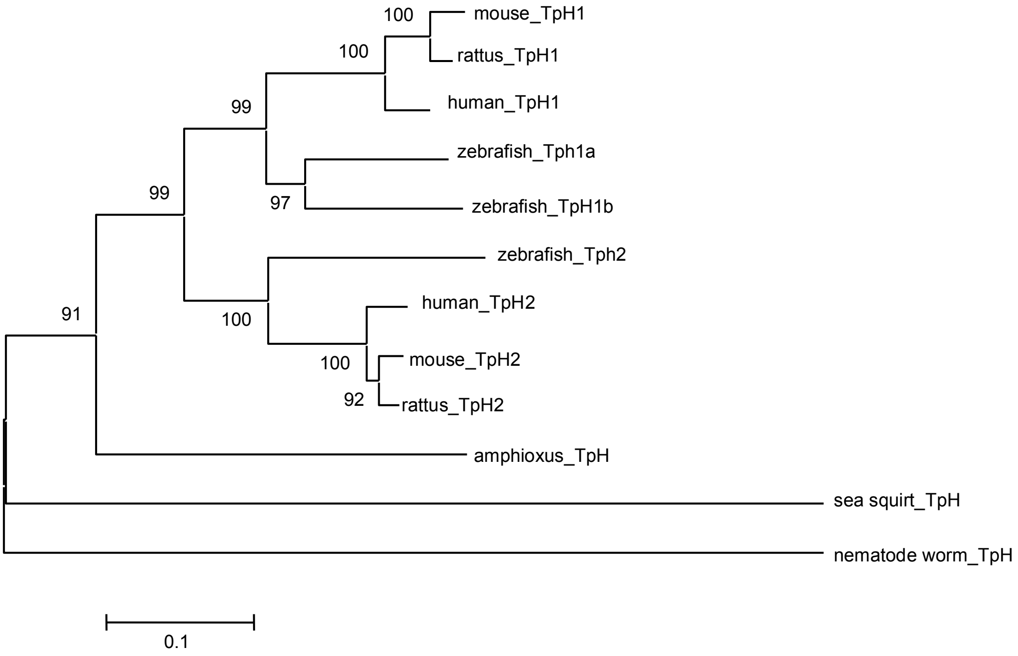

Phylogenetic trees inferred with Serotonin Transporter (SERT) proteins

ML

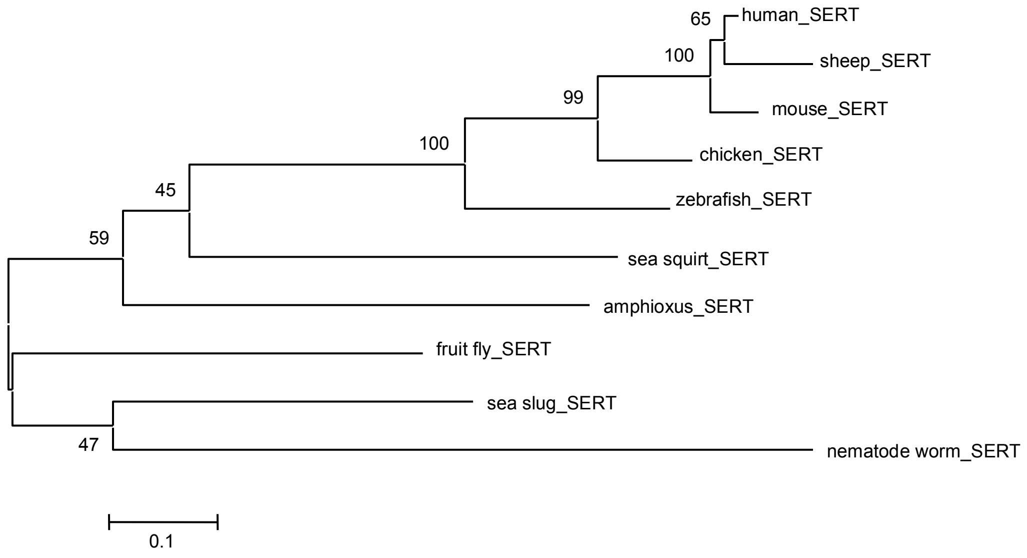

NJ

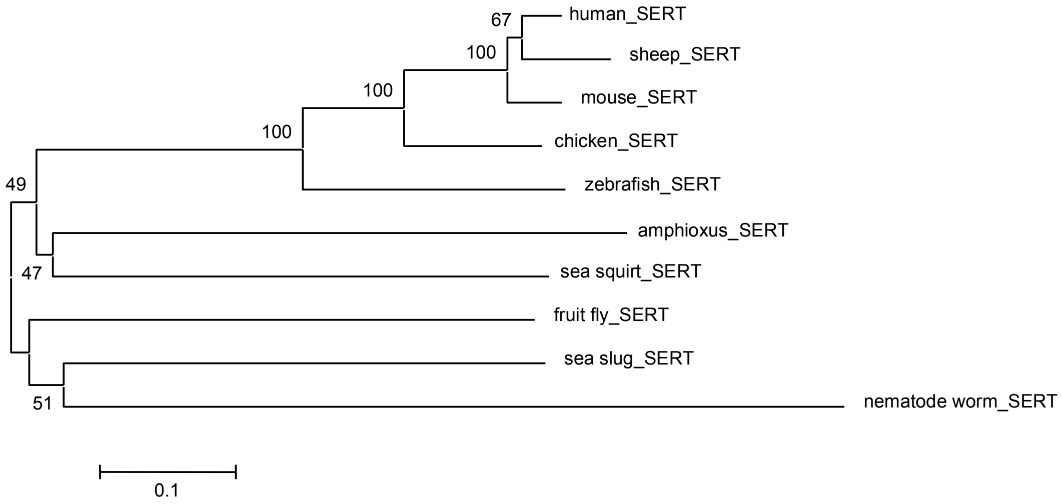

Phylogenetic trees inferred with Glutamic Acid Decarboxylase (GAD) proteins

ML

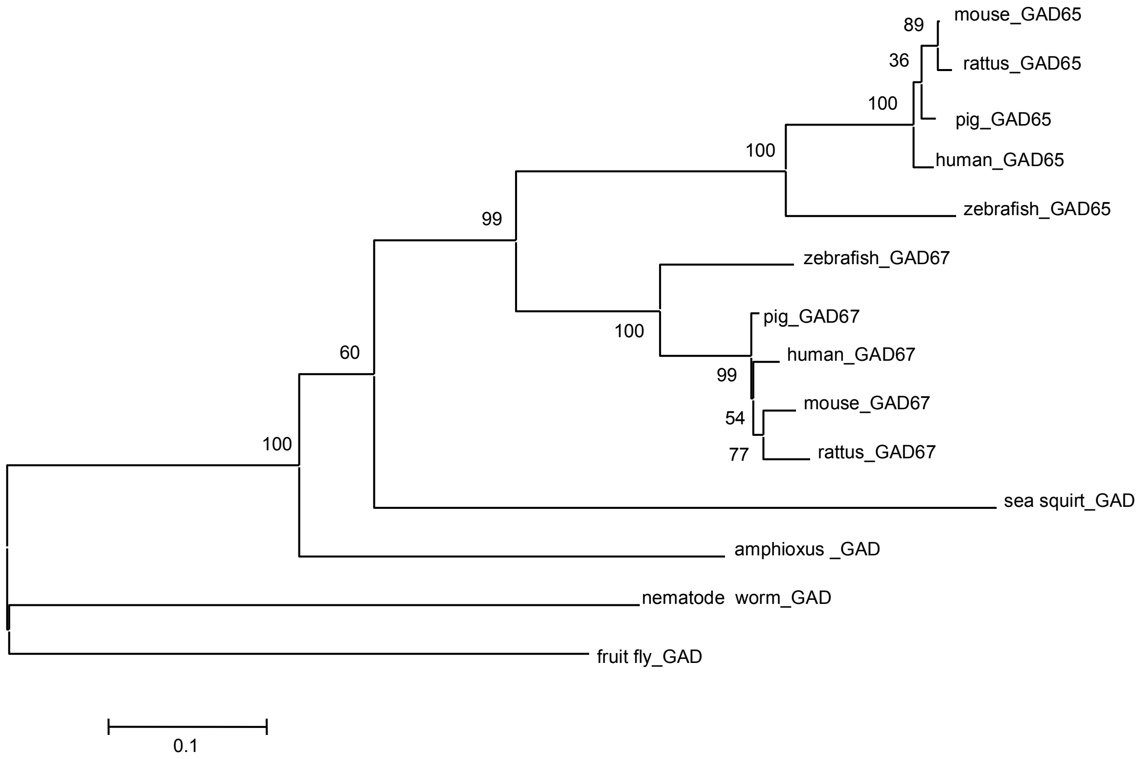

NJ

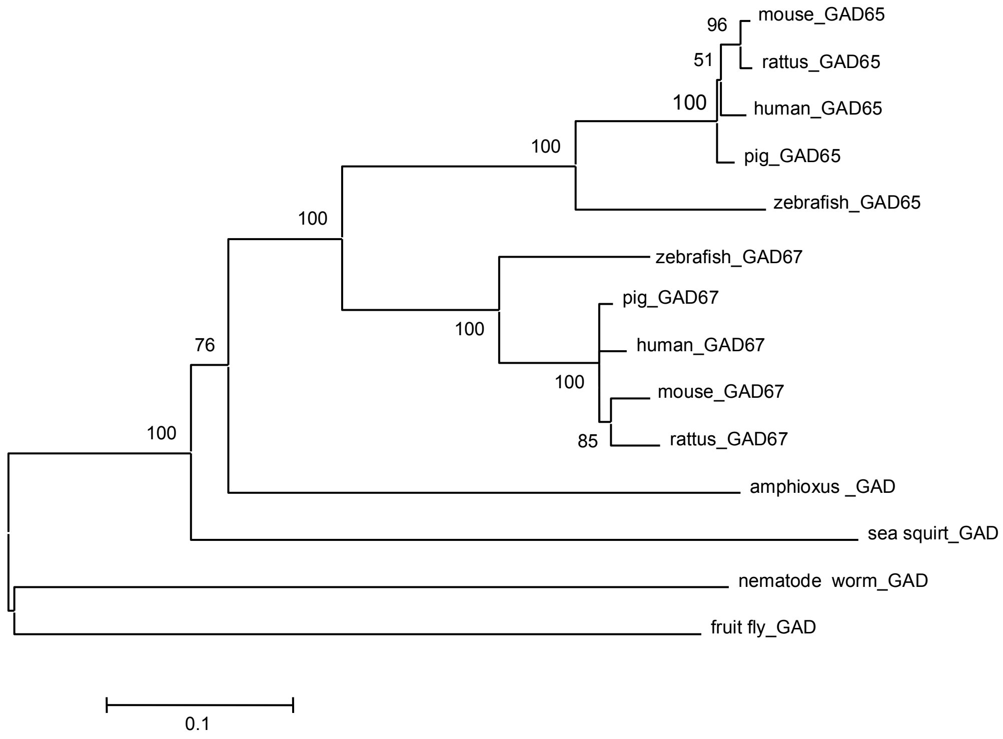

Phylogenetic trees inferred with Vesicular GABA/Glycine transporter (VGAT) proteins

ML

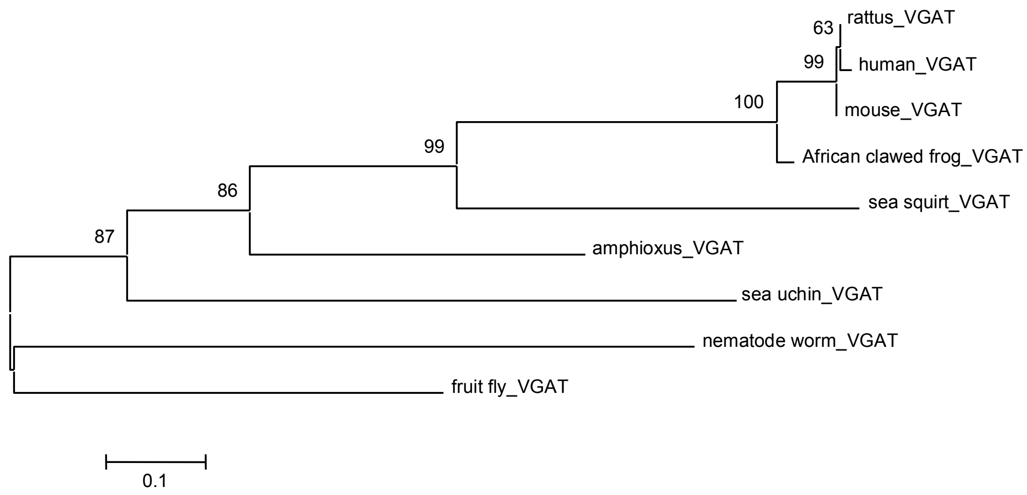

NJ

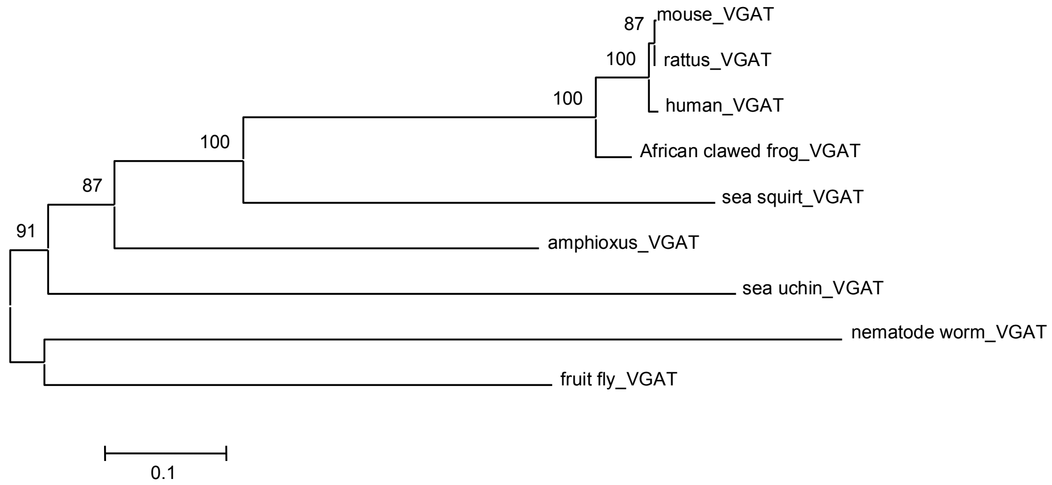

Supplement: Additional file 2 — Rooted phylogenetic trees of VGLUT, TpH, SERT, GAD and VGAT proteins. The results of both Maximum Likelihood (ML) and Neighbor Joining (NJ) analyses are shown. In each tree, the robustness of the phylogenetic branching patterns was assessed by bootstrap analyses in 1,000 resampling replicates. Taxonomic abbreviations are as follows: human (Homo sapiens), pig (Sus scrofa), sheep (Ovis aries), rat (Rattus norvegicus), mouse (Mus musculus), chicken (Gallus gallus), African clawed frog (Xenopus laevis), zebrafish (Danio rerio), amphioxus (Branchiostoma floridae), sea squirt (Ciona intestinalis), sea urchin (Strongylocentrotus purpuratus), sea slug (Aplysia californica), fruit fly (Drosophila melanogaster), nematode worm (Caenorhabditis elegans). [file 1471-2202-13-59-S2.pdf]
